# Genetic Architecture of Trans-Laminar Cribrosa Pressure Difference and Primary Open-Angle Glaucoma

**DOI:** 10.1167/iovs.67.4.51

**Published:** 2026-04-22

**Authors:** In-Shik Hong, Chamlee Cho, Beomsu Kim, Injeong Shim, Yeong Chan Lee, Sang-Hyuk Jung, Minku Song, Sanghyeon Park, Sanghoon Hong, Hyeonbin Jo, Hoyoung Kim, Je Hyun Seo, Hong-Hee Won

**Affiliations:** 1 Department of Digital Health, Samsung Advanced Institute for Health Sciences and Technology (SAIHST), Sungkyunkwan University, Samsung Medical Center, Seoul, Republic of Korea; 2 Division of Nephrology, Boston Children’s Hospital, Boston, Massachusetts, United States; 3 Kidney Disease Initiative, Broad Institute of MIT and Harvard, Cambridge, Massachusetts, United States; 4 Cardiovascular Disease Initiative, Broad Institute of MIT and Harvard, Cambridge, Massachusetts, United States; 5 Center for Genomic Medicine, Massachusetts General Hospital, Boston, Massachusetts, United States; 6 Department of Physiology, Ajou University School of Medicine, Suwon, Republic of Korea; 7 Department of Medical Informatics, Kangwon National University College of Medicine, Chuncheon, Republic of Korea; 8 Veterans Medical Research Institute, Department of Ophthalmology, Veterans Health Service Medical Center, Seoul, Republic of Korea

**Keywords:** glaucoma, intraocular pressure (IOP), genome-wide association study (GWAS), translaminar cribrosa pressure difference (TLCPD), risk factor

## Abstract

**Purpose:**

The purpose of this study was to investigate whether the genetic architecture of translaminar cribrosa pressure difference (TLCPD) provides genetic insights based on dual-pressure theory beyond intraocular pressure (IOP) and whether a TLCPD-based polygenic risk score (PRS) predicts primary open-angle glaucoma (POAG) risk and its pleiotropic effects.

**Methods:**

The genome-wide association study (GWAS) of TLCPD was conducted in 82,147 individuals of European ancestry from the UK Biobank (UKBB). Functional enrichment analysis and colocalization analysis were performed to identify associated genes, tissues, and pathways. PRS was calculated in an independent set of 268,734 unrelated European-ancestry individuals not included in the TLCPD GWAS. A survival analysis was utilized to examine the association between the PRS for TLCPD and the incidence of POAG. The phenome-wide association study (PheWAS) of PRS was applied to identify associations between TLCPD and other diseases.

**Results:**

We identified 77 independent loci, including 12 previously unreported loci. Enrichment and colocalization analyses identified candidate genes related to retinal cell death, including *SDCCAG8*, *PILRB*, *FBXO46*, *NUPR1*, and *JUND*, which were not previously associated with elevated IOP. Individuals in the top 1% of the PRS had a 4.48-fold higher risk of developing POAG than those in the bottom 20% (95% confidence interval = 3.70–5.43). PheWAS identified TLCPD PRS-associated traits including hypertension, glaucoma, obesity, arthropathies, and sleep apnea.

**Conclusions:**

This study identified TLCPD-associated variants, genes, cell types, tissues, and diseases in UKBB participants of European ancestry. These findings support the clinical relevance of TLCPD in glaucoma pathogenesis, implicating TLCPD-related genetic variants as contributor to POAG susceptibility.

Glaucoma is a progressive optic neuropathy characterized by the degeneration of retinal ganglion cells (RGCs) and their axons.^1^ Elevated intraocular pressure (IOP) and changes in the posterior displacement of the lamina cribrosa are putative features of primary open-angle glaucoma (POAG).^2^^–^^5^ However, its precise pathogenesis remains to be elucidated. As individuals with similar IOP levels may have different risks of developing the disease, other POAG risk factors, such as age, race, family history, and inadequate blood flow, may be involved in pathogenesis.^6^^–^^13^

Recently, cerebrospinal fluid pressure (CSFP) has been proposed as a potential risk factor for POAG.^14^^–^^17^ According to that theory, patients with POAG with normal pressure tend to have a lower CSFP than those without glaucoma.^18^^,^^19^ Conversely, patients with ocular hypertension have a greater CSFP than healthy controls.^20^

Translaminar cribrosa pressure difference (TLCPD), the difference between the CSFP and IOP, has been proposed to explain the susceptibility to POAG without elevated IOP,^14^^,^^19^^–^^24^ by reflecting the pressure gradient across the optic nerve head.^25^^,^^26^ TLCPD has been proposed to have an important influence on the optic nerve head, potentially beyond that explained by trans-corneal pressure difference (known as IOP) alone.^25^ However, measurement of CSFP is limited in the clinical management of glaucoma because lumbar puncture, the gold standard method for assessing CSFP, is invasive. In order to circumvent the invasive CSFP measurement, several estimation models have been proposed that rely on parameters obtainable through noninvasive means, including age, body mass index (BMI), and blood pressure (BP).^27^^,^^28^

TLCPD has been the subject of observational studies of glaucoma development with a limited number of subjects.^18^^–^^20^^,^^24^^,^^29^^–^^32^ Only a few studies have examined the association between TLCPD and glaucoma development using a prospective design.^19^^,^^30^^,^^31^^,^^33^^,^^34^ Although previous genome-wide association studies (GWASs) have identified more than 100 variants associated with IOP,^35^^–^^37^ no large-scale GWAS of TLCPD has been conducted. Given the growing interest in the roles of CSFP and TLCPD in the pathogenesis of glaucoma,^29^^,^^33^^,^^34^^,^^38^^–^^41^ investigating the genetic architecture of TLCPD is essential for a deeper understanding of glaucoma mechanisms.

To investigate the genetic architecture of TLCPD, we conducted a GWAS for TLCPD in 82,147 individuals of European ancestry from the UK Biobank (UKBB)*.* We calculated the polygenic risk score (PRS) for TLCPD and evaluated its potential to predict POAG and its pleiotropic effects.

## Methods

### Study Participants

The study protocol was approved by the Institutional Review Board of the Veterans Health Service Medical Centre (IRB No. 2022-03-034). Informed consent was waived due to the retrospective nature of the study, and all data were anonymized and de-identified. In addition, the UKBB committee approved the project (project ID: 96390) and the material transfer agreements. Analysis was conducted on participants of European ancestry from the UKBB study.

### Phenotype Definition

IOP (UKBB data fields 5263 and 5255), diastolic blood pressure (DBP; UKBB data fields 94 and 4079), BMI (UKBB data field 21001), and age (UKBB data field 21003) were used to estimate CSFP according to the Beijing iCOP regression formula (CSFP, millimeters of mercury [mm Hg] = 0.44 × BMI [kg/m²] + 0.16 × DBP [mm Hg] – 0.18 × age [years] – 1.91.^27^^,^^28^^,^^38^^,^^41^^–^^50^ The TLCPD of the participants was defined as the difference between IOP and CSFP (TLCPD = IOP – CSFP). Participants were excluded if they had extreme IOP values, reported taking IOP-related medications, or had a history of IOP-related surgeries (Supplementary Tables S1, S2). In total, 82,147 participants with TLCPD estimates were included in this study.

### Genome-Wide Association Analysis and Genetic Correlation

Associations between genomic variants and TLCPD were tested using SAIGE (version 1.1.3)^51^ in 82,147 quality-controlled participants of European ancestry with TLCPD estimates. This generalized linear mixed model was used to adjust for sample relatedness, with age, sex, genotyping batch, and the first 10 principal components (PCs) included as covariates. Variants that reached a genome-wide significance level (*P* < 5.0 × 10^−8^) were considered statistically significant.

### Cell Type-Specific Enrichment of Genes Mapped to GWAS Loci for a Given Complex Trait Using Seismic

We performed cell type-specific enrichment analysis using seismic^52^ with single-nucleus RNA-sequencing (snRNA-seq) retinal data^53^ to identify ocular cell types associated with cell type-specific gene expression mapped to the GWAS loci of TLCPD, POAG, and IOP. The analysis was conducted following the seismic framework.

### Colocalization With Expression Quantitative Trait Loci Database

Colocalization between GWAS and expression quantitative trait loci (eQTL) was performed using the coloc R software package.^54^ The eQTL summary statistics of retinal tissue from Ratnapriya et al. (EyeGEx)^55^ and Strunz et al.,^56^ (Supplementary Table S3) along with seven tissues from Genotype-Tissue Expression project (GTEx) version 8^57^ (aorta, coronary artery, tibial artery, cultured fibroblasts, left ventricle, kidney cortex, and whole blood) were used for colocalization analysis. We selected tissues regulating blood pressure or sharing mesodermal origin with ocular blood vessels.

### Functional Enrichment Analysis

We performed a functional enrichment analysis of the GWAS results with 36 categorized Medical Subject Heading (MeSH) terms using Data-driven Expression Prioritized Integration for Complex Traits (DEPICT; version 1 rel194)^58^ with default settings. Significant GWAS single-nucleotide polymorphisms (SNPs; *P* < 5.0 × 10^−8^) were used as input against a genetic background of SNPs derived from the 1000 Genomes Project phase III for the analysis.^59^ The results were considered significant after the false discovery rate (FDR) correction of <0.05.

### PRS Calculation

The PRS for TLCPD was calculated using the Bayesian genome-wide approach with the continuous shrinkage prior (PRS-CS) auto model.^60^ We calculated the PRS for TLCPD in an independent set of 268,734 unrelated European-ancestry individuals from the UKBB who were not included in the TLCPD GWAS due to unavailable TLCPD estimates.

### Survival Analysis With PRS and POAG Incidence

Among the 268,734 unrelated individuals with TLCPD PRS, survival analysis of PRS and POAG incidence was performed on 230,689 individuals with a censored date. Individuals were censored at the date of follow-up loss, date of follow-up end (July 31, 2021, for England; February 28, 2018, for Wales; and September 30, 2021, for Scotland), date of POAG diagnosis, or date of death, whichever came first.

### Phenome-Wide Association Study of PRS for TLCPD

A phenome-wide association study (PheWAS) of TLCPD PRS was conducted with a bias-reduced generalized linear model using the brglm R software package,^61^ adjusted for age, sex, and the first 10 PCs. To ensure sufficient statistical power, our PheWAS was restricted to 1117 of the 1645 phecodes mapped from UKBB participants’ International Classification of Diseases, Ninth Revision (ICD-9) and International Classification of Diseases, Tenth Revision (ICD-10) codes,^62^ excluding any phenotypes with fewer than 200 cases.^63^ Phenotypes significantly associated with TLCPD PRS were identified using a Bonferroni-corrected threshold (*P* < 0.05/1117).

Further details regarding the methodology and additional analyses are provided in the Supplementary Methods.

## Results

### Demographics of Study Participants and TLCPD

Among 408,188 UKBB participants of European ancestry, TLCPD was calculated for 82,147 individuals, with a mean of 4.02 mm Hg and a standard deviation of 5.02 mm Hg, due to the absence of necessary measurements in the remaining participants (Supplementary Tables S4, S5, Supplementary Figs. S1–S3). No evidence of significant population stratification was observed in study participants (Supplementary Figs. S4–S7).

### Identification of TLCPD-Associated Genomic Loci in a European Population

**Figure 1.**
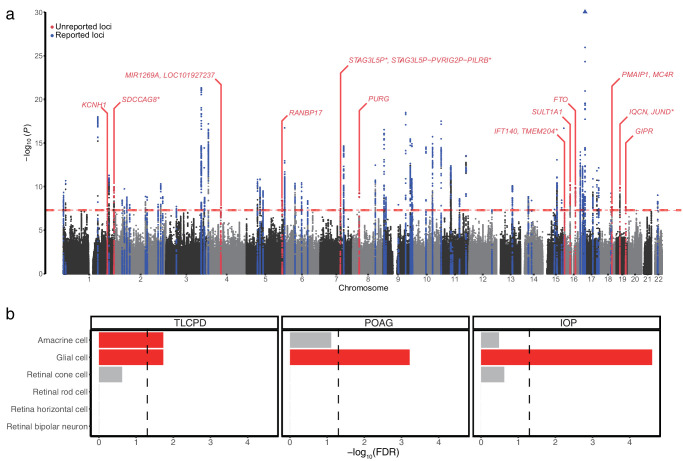
Genome-wide associations of TLCPD in UKBB participants of European ancestry. (**a**) Manhattan plot displaying −log_10_ (*P*) against the chromosomal position for TLCPD GWAS. *Red dashed line* represents the GWAS significance cutoff (*P* = 5 × 10^−8^). Independent genetic loci in *blue* and *red* represent previously reported and unreported TLCPD-associated loci, respectively. Nearest or colocalized (*asterisk*) genes in previously unreported loci are labeled. *Blue triangle* represents the most significant association, with a *P* value of 2.25 × 10^−31^. (**b**) Bar plot displaying −log_10_ (FDR) of the association between cell type and traits from seismic. *Dashed line* indicates the threshold for significance (false discovery rate [FDR] = 0.05), and each *red bar* represents a significant association that passed the FDR threshold. TLCPD, translaminar cribrosa pressure difference; POAG, primary open-angle glaucoma; IOP, intraocular pressure; UKBB, UK Biobank; GWAS, genome-wide association study; *P*, *P* value; FDR, false discovery rate.

GWAS was performed on 82,147 individuals of European ancestry from the UKBB to identify genomic loci associated with TLCPD. A total of 9,575,249 autosomal variants were tested for their associations with TLCPD using SAIGE (version 1.1.3),^51^ adjusted for age, sex, and the first 10 PCs (Fig. 1a, see Supplementary Fig. S7). We identified 77 independent genome-wide significant loci for TLCPD, including 12 loci that were not previously reported in IOP GWAS^35^^–^^37^^,^^64^^–^^72^ (see the Table, Supplementary Table S6). Although TLCPD is mathematically derived from IOP, BP, and BMI, 9 of the 12 loci have not been reported in previous large-scale GWAS for BP or BMI. Sensitivity analysis via a two-stage GWAS supported the robustness of our association results (Supplementary Table S7, Supplementary Methods). In these analyses, 73 of 77 lead SNPs remained significant (*P* < 6.49 × 10^−4^, Bonferroni correction for 77 tests) in meta-analyses of 10 randomly partitioned discovery and replication subsets. Of the 12 loci, 8 showed significance in the internal IOP GWAS conducted as part of this study (*P* < 6.49 × 10^−4^), whereas 4 loci (near *SULT1A1*, *FTO*, *PMAIP1*, and *GIPR*) were not associated with IOP (Supplementary Fig. S8, Supplementary Table S8). Six of the 77 lead variants were significant eQTLs of 36 genes across 42 tissues in the Genotype-Tissue Expression project GTEx^57^ (Supplementary Table S9). The SNP-based heritability of TLCPD was 0.25 (s.e. = 0.011) and the linkage disequilibrium score regression (LDSC) intercept^73^ of TLCPD was 1.07 (s.e. = 0.011) and sample-size scaled genomic inflation factor^74^ (λ_1000_ = 1.004; see Supplementary Fig. S7), indicated no significant population stratification. Consistent with this polygenic architecture, the polygenicity estimated from SBayesS was 1.72%, lies within the range of common physical measures previously reported (0.58%–5.03%).^75^ To estimate SNP-based genetic correlations,^76^ we conducted a GWAS of IOP in the identical participants of the TLCPD GWAS and obtained POAG GWAS summary statistics from Gharahkhani et al.^77^ TLCPD exhibited a significant positive genetic correlation (*r*_g_ = 0.50, s.e. = 0.039, *P* = 8.81 × 10^−38^), which was similar to the genetic correlation between IOP and POAG (*r*_g_ = 0.57, s.e. = 0.040, *P* = 4.45 × 10^−47^).

**Table.** Lead SNPs in Previously Unreported Loci Identified in the GWAS of TLCPD

rsID	GRCh37	Nearest Gene	Colocalized Gene	Annotation	Effect Allele	Other Allele	**β**	s.e.	*P* Value
rs35385507	1:211223493	*KCNH1*	−	Intronic	C	T	0.029	0.005	3.75 × 10^−8^
rs68192516	1:243485126	*SDCCAG8*	*SDCCAG8*, *CEP170*	Intronic	G	T	−0.033	0.005	1.13 × 10^−10^
rs35631117	4:67804304	*MIR1269A*, *LOC101927237*	−	Intergenic	G	T	−0.026	0.005	2.77 × 10^−8^
rs11747937	5:170633567	*RANBP17*	−	Intronic	A	G	0.029	0.005	3.94 × 10^−9^
rs182703269	7:99934453	*STAG3L5P*, *STAG3L5P-PVRIG2P-PILRB*	*PILRA*, *PILRB*, *STAG3L5P, STAG3L5P-PVRIG2P-PILRB*, *PMS2P1*, *ZCWPW1*, *MEPCE, TSC22D4*	ncRNA intronic	A	G	−0.033	0.006	3.67 × 10^−8^
rs10954772	8:30863938	*PURG*	−	Intronic	T	C	−0.031	0.005	5.89 × 10^−10^
rs8053605	16:1594158	*IFT140*, *TMEM204*	*TMEM204*, *JPT2*, *TELO2*	Intronic	A	G	−0.028	0.005	4.37 × 10^−8^
rs28676837	16:28627063	*SULT1A1*	*NUPR1*	Intronic	C	T	−0.031	0.005	6.81 × 10^−11^
rs1421085	16:53800954	*FTO*	*–*	Intronic	C	T	−0.030	0.005	1.36 × 10^−10^
rs12966550	18:57911330	*PMAIP1*, *MC4R*	*–*	Intergenic	G	A	−0.030	0.005	2.19 × 10^−8^
rs8109936	19:18388011	*IQCN*, *JUND*	*MAST3*, *JUND*	Intergenic	T	C	0.031	0.005	1.30 × 10^−10^
rs1800437	19:46181392	*GIPR*	*FBXO46*	Exonic	C	G	0.033	0.006	2.21 × 10^−8^

SNP, single-nucleotide polymorphism; GWAS, genome-wide association study; TLCPD, translaminar cribrosa pressure difference; GRCh37, chromosome and base pair position (GRCh37/hg19); β, coefficient of each SNP estimated by linear mixed model using SAIGE; s.e., standard error.

Twelve previously unreported lead SNPs were significantly associated with TLCPD (*n* = 82,147). Summary statistics for all independent genome-wide significant lead variants in the TLCPD GWAS are provided in Supplementary Table S6.

### Cell Type Trait Association of TLCPD

To identify the associations between cell types and GWAS results, we applied seismic^52^ to TLCPD, POAG, and IOP GWAS using cell type-specific expression from snRNA-seq of retina dissected from non-diseased human eyes^53^ (Fig. 1b, Supplementary Table S10). Among the cell types in the retina, amacrine cells (*P* = 6.18 × 10^−3^, FDR = 0.019) and glial cells (*P* = 4.69 × 10^−3^, FDR = 0.019) were significantly associated with TLCPD. Glial cells showed significant association with IOP (*P* = 4.16 × 10^−6^, FDR = 2.50 × 10^−5^) and POAG (*P* = 1.01 × 10^−4^, FDR = 6.05 × 10^−4^), whereas amacrine cells did not.

### Colocalization and Functional Annotation of the Identified TLCPD-Associated Loci

**Figure 2.**
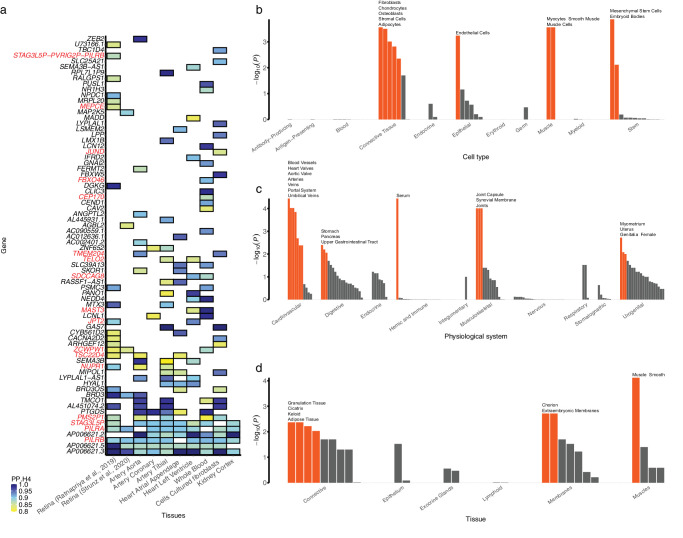
Colocalization of TLCPD-associated loci and eQTL in different tissues. (**a**) Heatmap of significant colocalization (PP.H4 ≥ 0.8) in eight tissues. The *x*-axis represents different tissues from GTEx, EyeGEx, and retinal eQTL data from Strunz et al., and the *y*-axis represents genes. *Red genes* represent colocalized genes in previously unreported loci. The color of each tile indicates the posterior probability of colocalization. (**b**) Cell type; (**c**) physiological system; and (**d**) tissue enrichment of TLCPD-associated loci. The height of the bar indicates the nominal log_10_(*P*) of enrichment from DEPICT. *Orange bars* indicate significant enrichment that passed the FDR correction (FDR < 0.05); these results are labeled above the bar plot. TLCPD, translaminar cribrosa pressure difference; IOP, intraocular pressure; GWAS, genome-wide association study; eQTL, expression quantitative trait loci; PP.H4, posterior probability for colocalization; *P*, = *P* value; FDR, false discovery rate.

The eQTLs for 74 genes from the retina and various GTEx tissues showed significant colocalization with TLCPD-associated variants (posterior probability for colocalization [PP.H4] ≥ 0.8; Fig. 2a, Supplementary Table S11). Of these 74 genes, 17 were colocalized with previously unreported loci (Supplementary Fig. S9). In the retinal tissue, 10 of the 17 colocalized genes showed significant colocalization, with the remaining 7 exhibited suggestive colocalization (PP.H4 > 0.5; Supplementary Fig. S10). Three genes were shared across GTEx, EyeGEx, and retinal eQTL from Strunz et al., whereas two genes were uniquely identified in EyeGEx.

### Tissue and Gene Set Enrichment of TLCPD GWAS

We identified cell types and tissues related to biological system functions, such as mesenchymal stem cells, blood vessels, and smooth muscle (Figs. 2b–d). In the functional enrichment test, the TLCPD GWAS was significantly enriched in 10 cell types, 17 physiological systems, and 7 tissues (Supplementary Tables S12–S14). Mesenchymal stem cells (*P* = 1.36 × 10^−4^), blood vessels (*P* = 3.64 × 10^−5^), and smooth muscle (*P* = 7.61 × 10^−5^) showed the most significant enrichment among cell categories, physiological systems, and tissue categories, respectively.

### Polygenic Risk Score for TLCPD and POAG Incidence Prediction

**Figure 3.**
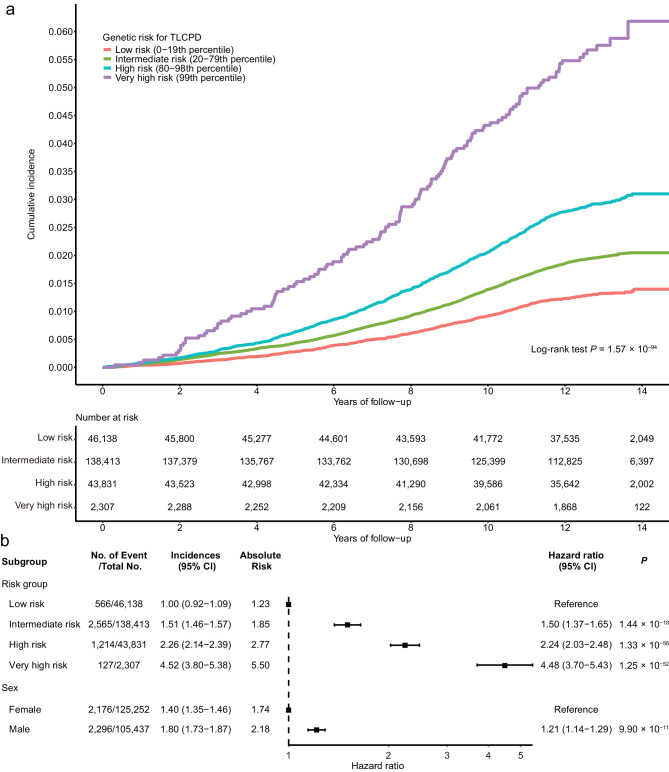
Survival analysis of POAG according to the polygenic risk group of TLCPD. (**a**) Kaplan-Meier curve for POAG incidence rates according to TLCPD PRS groups. (**b**) Forest plot of hazard ratio for POAG incidence according to TLCPD PRS group and sex. Cox proportional hazards regression model was adjusted for age, sex, genotyping array, and the first 10 principal components of ancestry, where the *dots* represent the estimated hazard ratio of each group. Each *horizontal error bar* represents the 95% confidence interval (CI) of the hazard ratio. Low-risk, 0 to 19th percentile; intermediate-risk, 20th to 79th percentile; high-risk, 80th 98th percentile; very high-risk, 99th to 100th percentile; POAG, primary open-angle glaucoma; TLCPD, translaminar cribrosa pressure difference; PRS, polygenic risk score; HR, hazard ratio; 95% CI, 95% confidence interval; *P*, *P* value.

During the median follow-up period of 12.25 years (interquartile range = 12.19–13.31 years), 1.9% of the 230,689 participants developed POAG (4472 patients with POAG and 226,217 controls) in 268,734 unrelated UKBB participants of European ancestry (Supplementary Table S15). Kaplan–Meier survival curves revealed that the increasing genetic risk for TLCPD was significantly associated with the risk of developing POAG during the follow-up period (log rank test *P* = 1.57 × 10^−94^). The risk of developing POAG was 4.48 times higher in the very high-risk group (top 1% of the TLCPD PRS) than in the low-risk group (bottom 20% of the TLCPD PRS; hazard ratio [HR] = 4.48, 95% confidence interval [CI] = 3.70–5.43; Fig. 3). The high-risk group (top 5% of the TLCPD PRS) and the intermediate-risk group (the second quintile, 20th to –40th percentile of the TLCPD PRS) showed 2.24-fold and 1.50-fold higher risks of POAG development, respectively, compared to the low-risk group.

### Phenome-Wide Association of PRS for TLCPD

**Figure 4.**
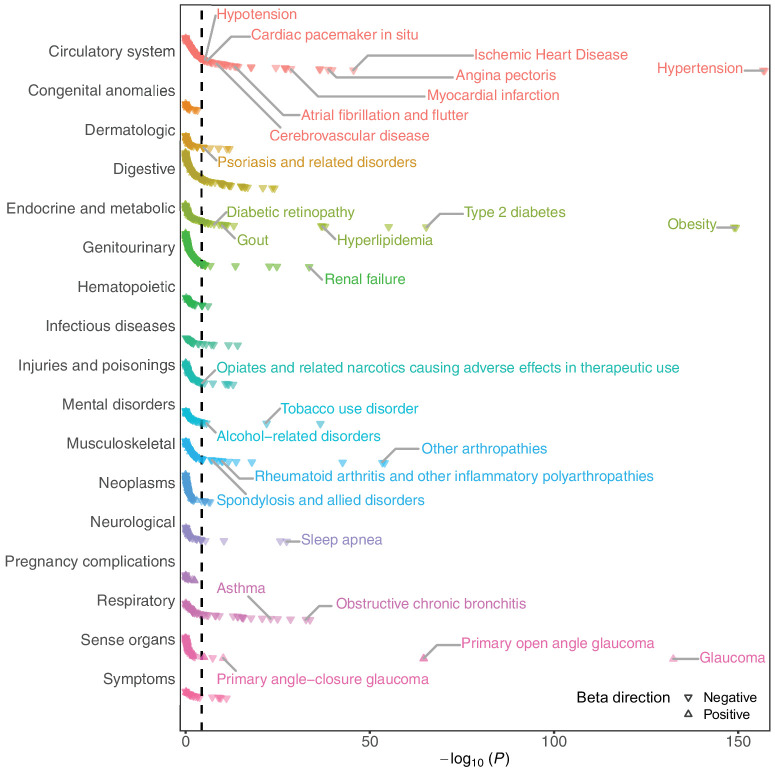
Phenome-wide associations of TLCPD PRS. PheWAS of TLCPD PRS for 1117 phecodes in UKBB participants of European ancestry. The *y*-axis represents 17 categories of phecodes and the *x*-axis represents the log_10_ of *P* values from the bias-reduced generalized linear regression, adjusting for age, sex, and the first 10 principal components of ancestry. *Triangular* and *inverted-triangular dots* indicate phenotypes with positive and negative effects for each trait, respectively. The *dashed vertical line* represents the significance threshold after applying Bonferroni correction (*P* < 0.05/1117). Clinically meaningful diseases in each category are labeled. PheWAS, phenome-wide association study; PRS, polygenic risk score; TLCPD, translaminar cribrosa pressure difference; UKBB, UK Biobank; *P*, *P* value; beta, effect size of TLCPD PRS for each phenotype estimated by bias reduction in binomial-response generalized linear models.

A total of 196 phenotypes, among 1117 disease phenotypes, were significantly associated with the TLCPD PRS in PheWAS after Bonferroni correction for multiple testing (*P* < 0.05/1117; Fig. 4, Supplementary Table S16). Essential hypertension was the most significantly associated phenotype (β = −0.13, s.e. = 0.005, *P* = 7.31 × 10^−158^), although open-angle glaucoma, whereas being the second most significant phenotype in the sense organ disease category, exhibited the largest effect size (β = 0.40, s.e. = 0.023, *P* = 2.36 × 10^−65^). Within the endocrine and metabolic category, “overweight, obesity, and other hyperalimentation” exhibited the largest effect size (β = 0.40, s.e. = 0.008, *P* = 4.58 × 10^−150^).

## Discussion

This GWAS identified 77 TLCPD-associated loci in 82,147 individuals of European ancestry from the UKBB, providing genetic evidence relevant to the dual-pressure theory of glaucoma. TLCPD may capture mechanical stress at the optic nerve head beyond IOP alone. Because direct CSFP measurement is invasive and impractical for large-scale cohorts, we applied a previously validated estimation formula^27^ as a pragmatic surrogate. Previous studies have reported mean differences in TLCPD between healthy and glaucoma groups.^30^^,^^78^ Building on this, our receiver operating characteristic curve analysis identified several candidate thresholds including 6.98 mm Hg based on the Youden index,^79^ 2.93 mm Hg for the true-positive rate of 0.85, and 11.68 mm Hg for the false-positive rate of 0.05 (see Supplementary Fig. S2, Supplementary Table S5). Whereas these values may serve as potential clinical thresholds, further validation is needed. Although recent discussions have raised important concerns regarding the accuracy and generalizability of this formula,^28^^,^^32^^,^^80^^–^^82^ previous validations show reasonable agreement with direct CSFP measurements.^83^^,^^84^ Specifically, Bland-Altman analysis by Jonas et al.^83^ and Kasahara et al.^84^ confirmed statistical agreement. Although the latter study had a relatively small sample size (*n* = 39) and Brazilian ethnic participants, the estimates fell within the 95% limits of agreement. Whereas the use of the estimated TLCPD may introduce phenotypic noise, our GWAS identified multiple genome-wide significant loci. These findings indicate that its use in a large-scale GWAS may be useful to examine the underlying biological signals.

We identified shared and divergent retinal cell types associated with TLCPD, POAG, and IOP. Colocalization and functional enrichment analyses prioritized 72 candidate genes across multiple tissues, including retinal tissue, highlighting genes such as *NPDC1* and *NUPR1*. Furthermore, individuals in the top 1% of the TLCPD PRS had a 4.48-fold higher risk of developing POAG than those in the bottom 20%, supporting the potential utility of TLCPD PRS for predicting POAG incidence. PheWAS revealed pleiotropic associations of TLCPD PRS with cardiometabolic and ocular traits, including hypertension, obesity, and glaucoma. SNP-based heritability of TLCPD was estimated to be 25%. TLCPD-associated loci showed a significant positive genetic correlation with POAG (*r*_g_ = 0.50, s.e. = 0.039), consistent with findings from previous clinical studies.^19^^,^^34^ Cell type-specific enrichment using seismic^52^ highlighted the cell type contributions to TLCPD, POAG, and IOP. Glial cells were significantly associated with TLCPD, POAG, and IOP. This finding is consistent with previous evidence linking glial cells to glaucomatous neurodegeneration.^85^^–^^88^ Amacrine cells showed a significant association exclusively with TLCPD. These results suggest the genomic loci associated with TLCPD, POAG, and IOP may underlie both shared and trait-specific mechanisms. Although this finding generates a hypothesis consistent with known links between optic nerve sheath fenestration and amacrine cells,^89^ our reliance on retina-derived data necessitates further validation using data of optic nerve head or lamina cribrosa-specific tissues.

Several genes located near previously unreported TLCPD-associated loci, which were not significant in prior IOP GWAS, have been implicated in glaucoma susceptibility in previous studies. Mutations in *SDCCAG8* are associated with retinal ciliopathies via the *RPGRIP1* interactome.^90^^,^^91^ *RPGRIP1*, encoding a vital retinal signaling scaffold, was proposed as a functional candidate gene for glaucoma.^92^ Consistent with these findings, prior glaucoma GWAS highlighted the *SDCCAG8* locus for further investigation.^93^ *PMAIP1*, encoding the pro-apoptotic protein *Noxa*, has been reported to induce RGC apoptosis following *NMNAT1* downregulation, suggesting a potential role in glaucoma-associated neurodegeneration.^94^^,^^95^ *MC4R* is known to mediate the effects of α-melanocyte-stimulating hormone, thereby suppressing retinal cell death, while restoring visual function in the retina.^96^^,^^97^ *JUND*, a member of the AP-1 transcription factor family, has been implicated in RGC responses to axonal injury through stress-activated transcriptional programs involved in neuronal degeneration.^98^

Genes associated with TLCPD identified through colocalization analysis in retinal tissue may represent candidates for future functional studies exploring the potential role of TLCPD in glaucoma. *NPDC1* has been reported as a crucial participant in retinal progenitor cell proliferation and differentiation which may affect optic neuropathy.^99^ *ZNF652*, a blood pressure-associated gene identified in a previous GWAS,^100^ is involved in the transforming growth factor beta signaling cascade, a key pathway in glaucoma pathogenesis.^101^ *MRPL20*, a DBP-associated gene identified in a previous study,^102^ is downregulated in glaucomatous vitreous cells^103^ and cultured human trabecular meshwork tissue.^104^ *NUPR1*, induced under diverse cellular stresses,^105^^,^^106^ has been shown to promote the activation of the anti-apoptotic protein Bcl-xL.^106^^,^^107^ It has been proposed as a novel therapeutic target for preventing RGC death following axonal injury.^108^ Although our findings are based on retina-derived data and therefore cannot fully capture the biomechanics at the optic nerve head or lamina cribrosa, these genes may reflect a biological link between genetic susceptibility to the dual-pressure gradient and differences in RGC vulnerability. Therefore, they may represent candidate targets for dissecting glaucoma pathobiology, warranting further experimental validation.

Previous studies have identified associations between the incidence of glaucoma and genetic risk scores, primarily focusing on IOP, vertical cup-to-disc ratio, and POAG itself.^36^^,^^67^^,^^109^^–^^119^ In this study, the polygenic risk of TLCPD was associated with the incidence of POAG in UKBB participants of European ancestry, suggesting that the genetic pathways underlying TLCPD may contribute to POAG pathogenesis. Individuals in the top 1% of the TLCPD PRS group exhibited a 4.48-fold higher risk of developing POAG than those in the bottom 20% of the TLCPD PRS group. Incorporating traditional clinical risk factors into TLCPD estimation may enhance its clinical relevance and implications.

The PRS of TLCPD was associated with 196 of 1117 diseases in UKBB participants of European ancestry. The inverse association between TLCPD PRS and hypertension in the PheWAS may be explained by the inclusion of DBP in the CSFP estimation formula applied in this study. The negative association between TLCPD PRS and obesity is consistent with the positive correlation between body fat accumulation and CSFP.^120^^–^^122^ The negative association between TLCPD PRS and sleep apnea was further supported by a previous study showing that sleep apnea can lead to increased intracranial pressure.^123^ The positive association between TLCPD PRS and glaucoma is consistent with previous studies^14^^,^^19^^–^^24^ and aligns with the findings of the survival analysis conducted in the current study. Furthermore, these pleiotropic associations highlight the importance of considering a broad range of health conditions and related disorders to better understand the underlying pathways of TLCPD.

Our study has several limitations. First, our study was conducted on UKBB participants of European ancestry aged 40 to 69 years at baseline. Additionally, the GWAS was limited to individuals with complete IOP, BP, and BMI data, whereas the PRS group consisted of independent individuals excluded from the GWAS due to the lack of such clinical data. Although we observed negligible differences in the distribution of obesity, hypertension, and glaucoma between the GWAS and PRS groups (Cohen's φ < 0.1; see Supplementary Fig. S4), our sample may not fully represent the general population. Second, relying on code-based definitions for POAG limited our ability to assess symptom severity, subtype classification, and specific treatment details and may misclassify patients. This lack of granular phenotypes warrants caution when interpreting incidence models and effect sizes. Third, the estimated TLCPD cannot fully capture the complex, dynamic nature of CSFP, such as posture and diurnal variations, potentially introducing non-differential error or systematic bias. Fourth, direct replication in independent cohorts with relevant phenotypic data^124^^–^^126^ was not feasible due to restricted individual-level data access. To compensate and mitigate the risk of false-positive results, we conducted internal 10-fold cross-validation. Therefore, future replication studies in independent populations are needed. Finally, the functional relevance of the identified TLCPD-associated loci has not been experimentally validated in vitro or in vivo. Moreover, functional annotations derived from retina data may not fully capture the complex biology of the optic nerve head, lamina cribrosa, or CSFP. Therefore, our findings should be interpreted as hypothesis-generating rather than definitive mechanistic conclusions.

Despite these limitations, this study has several key strengths. To the best of our knowledge, this is the first GWAS of estimated TLCPD. Our study provides insights into the shared and divergent genetic architectures of TLCPD, POAG, and IOP by leveraging a large-scale population cohort that includes integrated IOP measurements and clinical records. Another strength is the TLCPD PRS assessment as a risk factor for POAG incidence and its pleiotropic associations, highlighting the importance of considering diverse phenotypes to gain a deeper understanding of the complex etiology and underlying TLCPD pathways in POAG.

In conclusion, we identified TLCPD-associated variants, genes, cell types, tissues, and diseases in UKBB participants of European ancestry. Our results offer fundamental insights into the genetic architecture of TLCPD, advancing our understanding of TLCPD-mediated glaucoma pathogenesis.

## Supplementary Material

Supplement 1

Supplement 2
