# Supplementary material for: Genetic Architecture of Trans-Laminar Cribrosa Pressure Difference and Primary Open-Angle Glaucoma
Source: Invest Ophthalmol Vis Sci. 2026 Apr 22;67(4):51. doi: 10.1167/iovs.67.4.51 (PMC13107994; doi:10.1167/iovs.67.4.51)
Supplement: Supplement 1 [file iovs-67-4-51_s001.docx]

**Supplementary Material**

**Supplementary Methods**

*Phenotype Definition*

Ophthalmic assessment was introduced as an enhancement in 2009 for six assessment centers located across the UK, including Liverpool and Sheffield in North England, Birmingham in the Midlands, Swansea in Wales, and Croydon and Hounslow in Greater London. IOP of the UKBB participants was measured once in each eye using an Ocular Response Analyzer (ORA; Reichert) as the corneal compensated IOP. Participants who had undergone eye surgery within the past four weeks or had an eye infection were excluded from the study. The ORA is a non-contact tonometer that measures the force required to flatten the cornea using an air jet. Goldmann-correlated IOP (IOPg) was derived by calibrating the average of the two pressures. IOPg is the most frequently used measurement in clinical practice and is the gold standard for IOP measurement. The IOP of the study participants was calculated as the maximum value between the right and left eyes for each participant with available data for both eyes. If data were available for only one eye, we considered the value to be the participant's IOPg. For the measurement of DBP (UKBB data fields 94 and 4079), the mean of two automated blood pressure readings was used, with manual readings imputed in the absence of automated reading data. We excluded individuals whose IOP levels may have been altered from physiological norms owing to non-genetic causes. Only data on regular treatments were included in the database (UKBB data field 20003); data on short-term medication use were not collected. Individuals who reported taking IOP-related medications (Supplementary Table S15) were excluded. Surgical data were obtained from the hospital inpatient data (UKBB data field 41272). Individuals with a history of IOP-related surgeries (Supplementary Table S16) were excluded. Individuals with IOPg below 5 mmHg or above 60 mmHg were excluded to account for extreme values. To investigate TLCPD and dual pressure theory, two pragmatic^1,2^ surrogate estimation was available. To mitigate calibration and minimize bias, we compared two methods. As illustrated in Supplementary Fig. S3, CSFP distribution derived from the Mayo^2^ formula deviated from established clinical knowledge in our study population. In contrast, the Beijing formula^1^ exhibited a distribution more closely aligned with clinical expectations. Therefore, we selected the Beijing formula^1^ for this study.

*Genotyping, Quality Control, and Imputation*

UKBB participants (version 3; March 2018) were genotyped for over 800,000 SNPs using either the Affymetrix UK BiLEVE Axiom array or the Affymetrix UK Biobank Axiom array. Genotyped data were phased using SHAPEIT3^3^ and imputed using IMPUTE2^4^ with the merged 1000 Genomes Project phase 3 panel and the UK 10 K panel. Following imputation, variant level quality control (QC) was performed by filtering SNPs on four criteria: (1) minor allele frequency < 5 × 10^−3^, (2) imputation quality score < 0.4, (3) call rate < 95%, and (4) Hardy–Weinberg equilibrium *P* < 1 × 10^−6^. In total, 9,575,249 imputed autosomal SNPs met the QC criteria. Sample-level QC was conducted by excluding samples based on three criteria: (1) participants identified as not of ‘White British’ ancestry according to either self-report or PCA of genetic ancestry, (2) mismatched sex, and (3) participants who withdrew from the UKBB. To validate the robustness of the PCs in addressing population stratification, we conducted an additional PCA using unrelated European participants from the UKBB who identified as ‘White British’ (UKBB data field 21000). These PC loadings were applied to compute the PCs for all TLCPD-estimated participants. The top 10 PCs from this process were incorporated as covariates in the downstream analyses.

*Lead variants and Genetic Architecture*

To identify the independent lead variants, stepwise selection was conducted for the significant GWAS results using GCTA-COJO (v.1.91.2).^5^ The scaled genomic inflation factor (λ_1000_) adjusted for a sample size of 1,000 was calculated with this formula (1+1000(𝜆−1)/n).^6^ SNP-based heritability and genetic correlations were estimated using LDSC (v.1.0.1)^7,8^ with the reference LD scores of the European ancestry population from the 1000 Genomes Project phase 3.^9^ The GWAS for IOP was conducted in the same participants as the TLCPD GWAS to estimate the genetic correlation with POAG. Summary statistics of the POAG GWAS were obtained from Gharahkhani et al. (2021).^10^

*Calculation of polygenicity*

To assess the polygenic architecture of TLCPD, we applied SBayesS,^11^ a summary-level Bayesian mixed linear model, to estimate polygenicity (the proportion of SNPs with non-zero effects) using GWAS summary statistics and a linkage disequilibrium (LD) reference panel. We used the precomputed banded LD matrix with a 3 cM window size, based on 10,000 European individuals from UK Biobank, obtained from the SBayesS website. This approach was adopted as it has been shown to improve prediction accuracy^12^ and the analysis was performed using default parameters.

*Sensitivity analysis*

As a sensitivity analysis, we conducted a two-stage GWAS to examine whether our association results were robust. We randomly divided the 82,147 European samples into 10 subsets. One subset was used as a replication set, and the remaining nine subsets were used as a discovery set, creating 10 pairs of discovery and replication sets. For the 77 lead SNPs, we conducted an association test in a discovery set and a replication set and performed a meta-analysis of the results from the discovery set and replication set. We repeated this process for all possible 10 combinations. Statistical significance was evaluated using a Bonferroni-corrected threshold (*P* < 0.05/77)

*Cell Type-Specific Enrichment of Genes Mapped to GWAS Loci for a Given Complex Trait Using Seismic*

The analysis was conducted following the seismic framework: (i) mapping GWAS results to genes using MAGMA (v.1.10)^13^; (ii) calculating specificity scores in the snRNA-seq data using seismic; and (iii) quantifying cell type trait associations through linear regression between Z-scores from MAGMA and specificity scores. Seismic analysis assumes that the genes that best characterize a cell type are not necessarily those most highly expressed in that cell type, but rather those that are most specific to it

*Colocalization criteria*

Colocalization with eQTL data was conducted for variants within 500 kb of each GWAS lead variant. For reliability, results with a high posterior probability for colocalization (PP.H4 ≥ 0.8) were considered significant, whereas those with PP.H4 > 0.5 were deemed suggestively colocalized for retinal tissue. PP.H4 is the posterior probability for hypothesis H_4_, defined as the colocalized signal between a significant GWAS association and a significant eQTL association.

*Survival Analysis with PRS and POAG Incidence*

A total of 4,472 patients with POAG were defined as those who (i) received an ICD-10 diagnosis of ‘primary open-angle glaucoma’, ‘other glaucoma’, or ‘glaucoma, unspecified’; (ii) received an ICD-9 diagnosis of ‘open-angle glaucoma’, or ‘glaucoma, unspecified’; and (iii) did not receive a diagnosis of primary angle-closure glaucoma (PACG). The date of POAG diagnosis was defined as the earliest date of the ICD diagnosis. Of the remaining individuals, 226,217 were defined as controls, characterized by the absence of ICD-9 diagnostic codes (365.0, 365.1, 365.2, 365.5, 365.6, and 365.9) and ICD-10 diagnostic codes (H40.0, H40.1, H40.2, H40.3, H40.4, H40.5, H40.6, H40.8, and H40.9). Additionally, individuals with self-reported glaucoma or those who did not respond to the eye problem questionnaire (UKBB data fields 20002 and 6148) were excluded from the control group. The association between the PRS for TLCPD and POAG incidence was investigated using Kaplan–Meier curves and Cox proportional hazard regression models. The study participants were categorized into four risk groups based on the TLCPD PRS: low (0–19%), intermediate (20–79%), high (80–98%), and very high (99–100%). The log-rank test was used to compare the Kaplan-Meier curves between groups. The Cox proportional hazards regression model was adjusted for age, sex, genotyping array, and the first 10 PCs.

**Supplementary Figures**

**Supplementary Fig. S1.** Flowchart for TLCPD outcome variables derivation for UKBB GWAS, TLCPD PRS, and survival analysis.

**Supplementary Fig. S2.** ROC curve analysis of TLCPD for discriminating glaucoma from healthy controls.

**Supplementary Fig. S3.** Phenotype distribution of GWAS group (n = 82,147) and PRS group (n = 268,734).

**Supplementary Fig. S4.** Comparison of phenotypic distributions between the GWAS (n = 82,147) and PRS (n = 268,734) groups.

**Supplementary Fig. S5.** Principal component analysis (PCA) of genetic variants in UKBB participants of genetically defined European ancestry.

**Supplementary Fig. S6.** Principal component analysis (PCA) of genetic variants in 82,147 TLCPD-estimated UKBB participants of European ancestry.

**Supplementary Fig. S7.** Quantile-quantile plot for GWAS of TLCPD (n = 82,147).

**Supplementary Fig. S8.** Regional plots of previously unreported loci in the TLCPD GWAS (top) and IOP GWAS (bottom).

**Supplementary Fig. S9.** Regional plots of previously unreported and colocalized loci from TLCPD GWAS (top) and eQTLs of the colocalized gene (bottom).

**Supplementary Fig. S10.** Bar plot of PP.H4 values in retinal tissues for genes colocalized with unreported loci.

**Supplementary Tables**

**Supplementary Table S1.** List of IOP-related medications.

**Supplementary Table S2.** List of IOP-related operations.

**Supplementary Table S3.** Information on retinal eQTL data used in colocalization analysis.

**Supplementary Table S4.** Characteristics of study participants in the TLCPD discovery set.

**Supplementary Table S5.** Operating points of TLCPD in discriminating glaucoma based on ROC analysis.

**Supplementary Table S6.** Summary statistics of the 77 lead variants identified in the TLCPD GWAS.

**Supplementary Table S7.** Two-stage TLCPD GWAS results for 10 randomly divided data sets.

**Supplementary Table S8.** Previously reported IOP GWAS significant loci from the GWAS catalog.

**Supplementary Table S9.** eQTL associations of lead variants in 12 previously unreported loci in the TLCPD GWAS from the GTEx (*P* < 5e-08).

**Supplementary Table S10.** Association between retinal cell types and TLCPD GWAS, POAG GWAS, and IOP GWAS.

**Supplementary Table S11.** Colocalization between GWAS results and eQTL in multiple tissues.

**Supplementary Table S12.** Cell enrichment analysis using DEPICT for TLCPD-associated loci.

**Supplementary Table S13.** Physiological system enrichment analysis using DEPICT for TLCPD-associated loci.

**Supplementary Table S14.** Tissue enrichment analysis of TLCPD-associated loci using DEPICT.

**Supplementary Table S15.** Baseline characteristics of participants according to the PRS categories for TLCPD.

**Supplementary Table S16.** Summary statistics for 196 significant PheWAS results of the TLCPD PRS.

**Supplementary Figures**

**
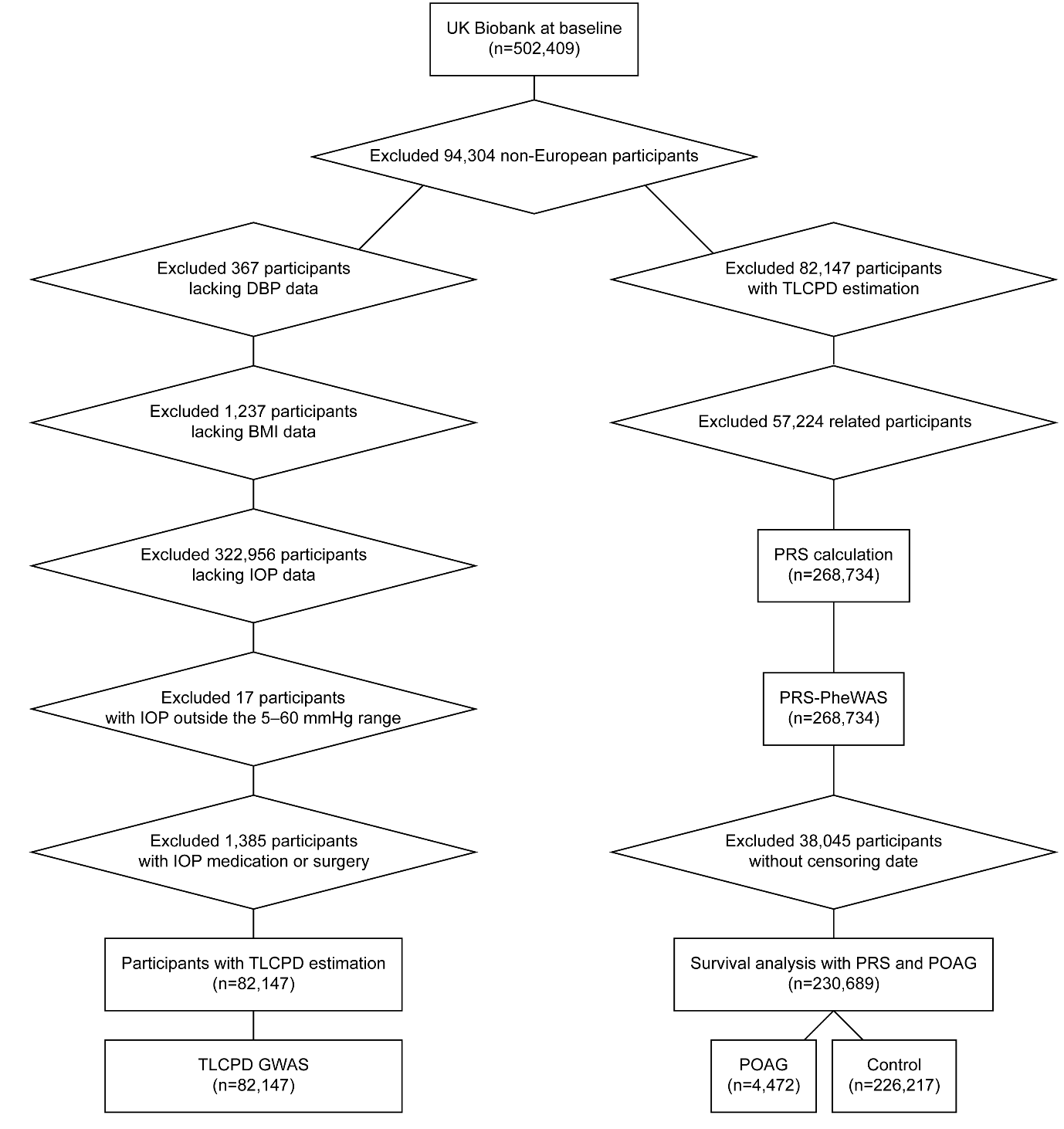
**

**Supplementary Fig. S1.** Flowchart for TLCPD outcome variables derivation for UKBB GWAS, TLCPD PRS, and survival analysis. Rhomboid nodes represent the number of individuals excluded for not meeting specific criteria; rectangular nodes represent the number of individuals included in the analysis. EUR, European; DBP, diastolic blood pressure; TLCPD, trans-laminar cribrosa pressure difference; UKBB, UK Biobank; GWAS, genome-wide association study; BMI, body mass index; IOP, intraocular pressure; PRS, polygenic risk score; PheWAS, phenome-wide association study; POAG, primary open-angle glaucoma.


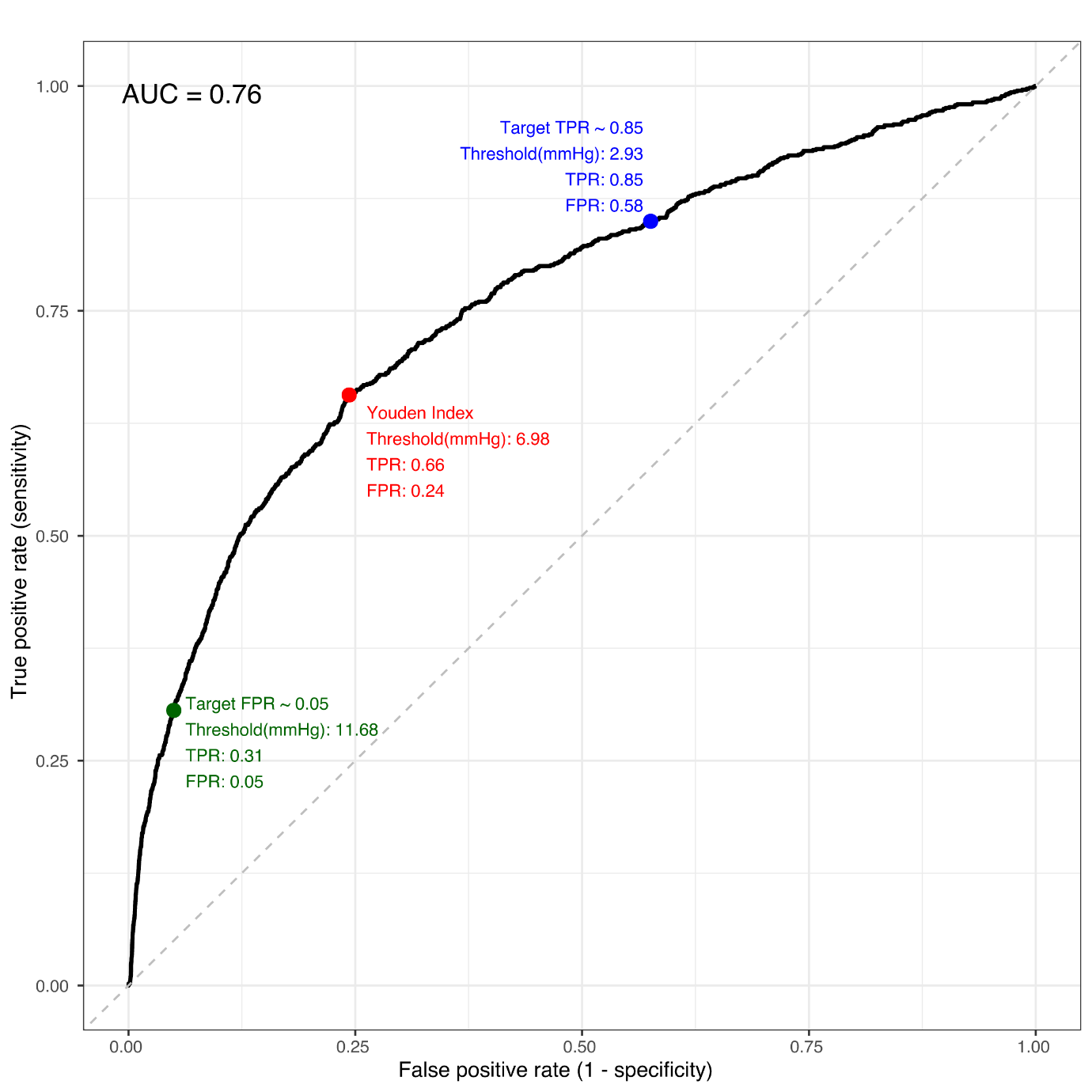


**Supplementary Fig. S2.** ROC curve analysis of TLCPD for discriminating glaucoma from healthy controls. The ROC curve illustrates the diagnostic performance of TLCPD (AUC = 0.76). The red circle represents the operating point determined by the maximum Youden index (threshold = 6.98 mmHg). The green and blue circles indicate the operating points at FPR of 0.05 (threshold = 11.68 mmHg) and TPR of 0.85 (threshold = 2.93 mmHg), respectively. ROC, receiver operating characteristic; TLCPD, translaminar pressure difference; AUC, area under the curve; FPR, false positive rate; TPR, true positive rate.

**
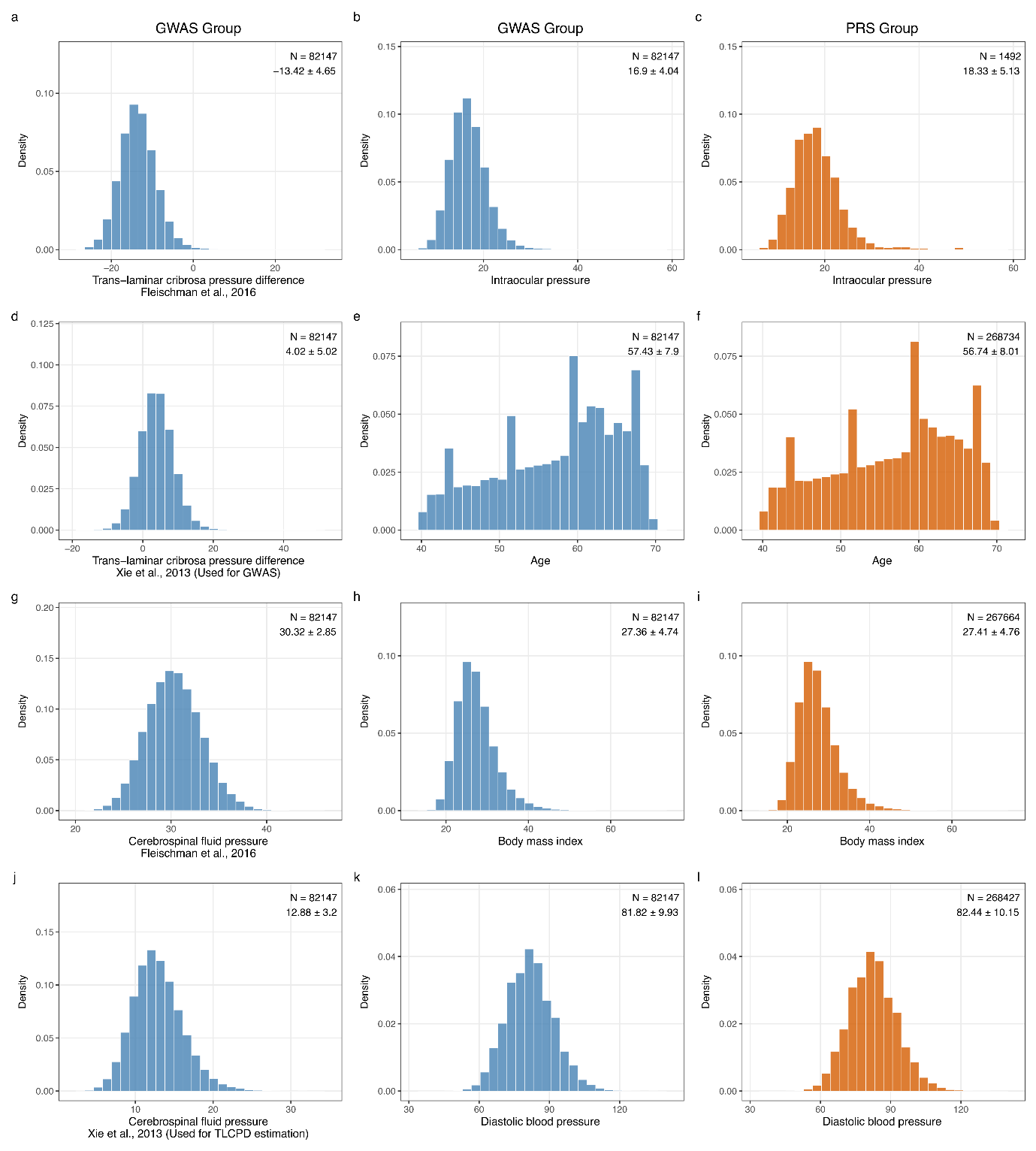
**

**Supplementary Fig. S3.** Phenotype distribution of GWAS group (n = 82,147) and PRS group (n = 268,734). **a-d,** distributions of two different estimations for CSFP and TLCPD in the GWAS group. **e-h,** distributions of measured phenotypes in the GWAS group. **i-l,** distributions of the corresponding measured phenotypes in the PRS group. Histogram shows the density of each trait. IOP, intraocular pressure; BMI, body mass index; TLCPD, translaminar pressure difference; CSFP, cerebrospinal fluid pressure.


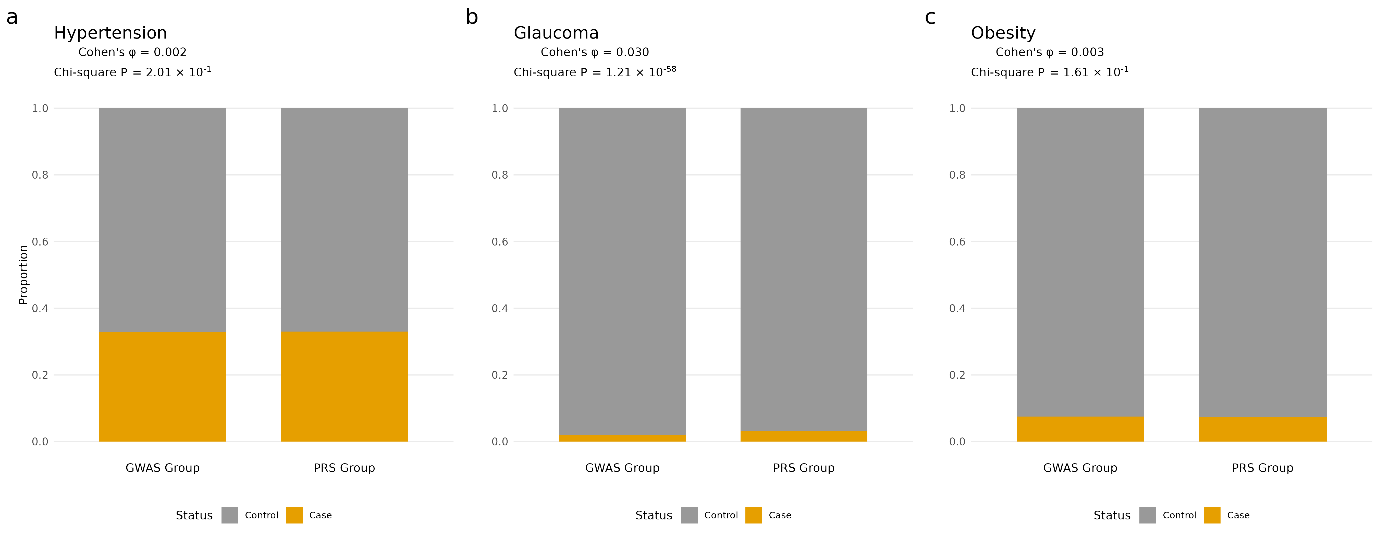


**Supplementary Fig. S4.** Comparison of phenotypic distributions between the GWAS (n = 82,147) and PRS (n = 268,734) groups. Stacked bar plots show the proportions of cases (orange) and controls (gray) for three representative phenotypes. **a,** hypertension; (phecode 401) **b,** glaucoma (phecode 365) **c,** obesity (phecode 278.1).

**
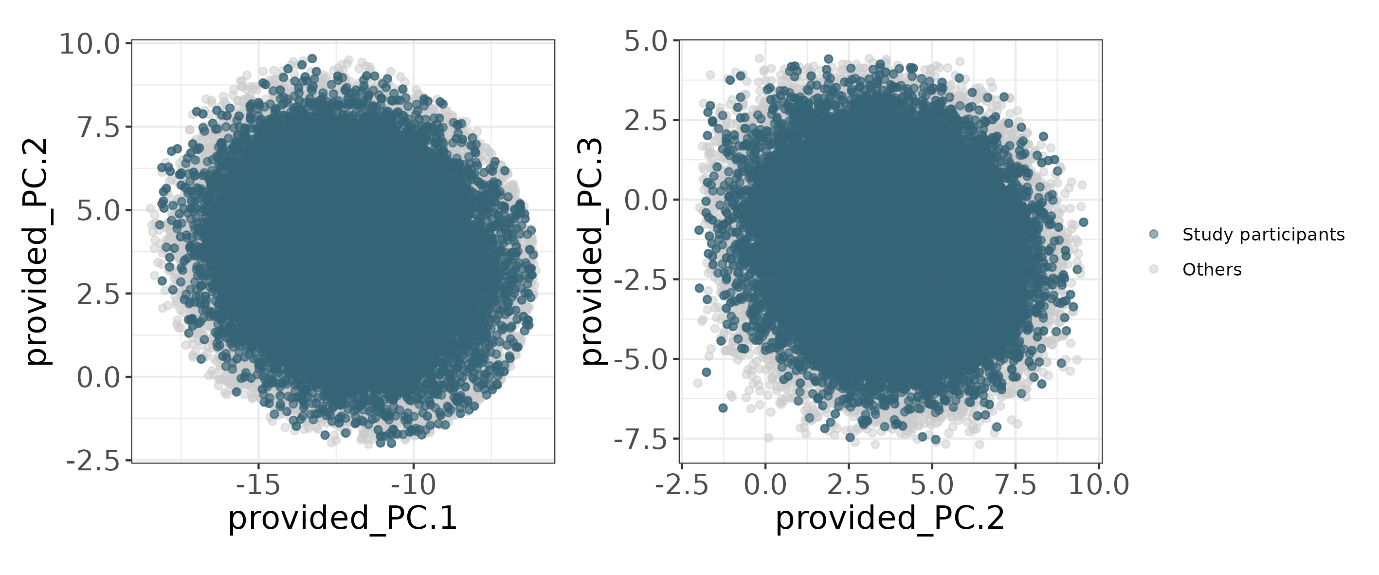
**

**Supplementary Fig. S5.** Principal component analysis (PCA) of genetic variants in UKBB participants of genetically defined European ancestry. The first three principal components of genetic ancestry provided by the UKBB (UKBB data field 22009) are represented. Each dot represents a UKBB participant of genetically defined European ancestry (UKBB data field 22006) with (teal) or without (gray) TLCPD estimates. PC, principal component; PCA, principal component analysis; UKBB, UK Biobank; EUR, European; TLCPD, trans-laminar cribrosa pressure difference.


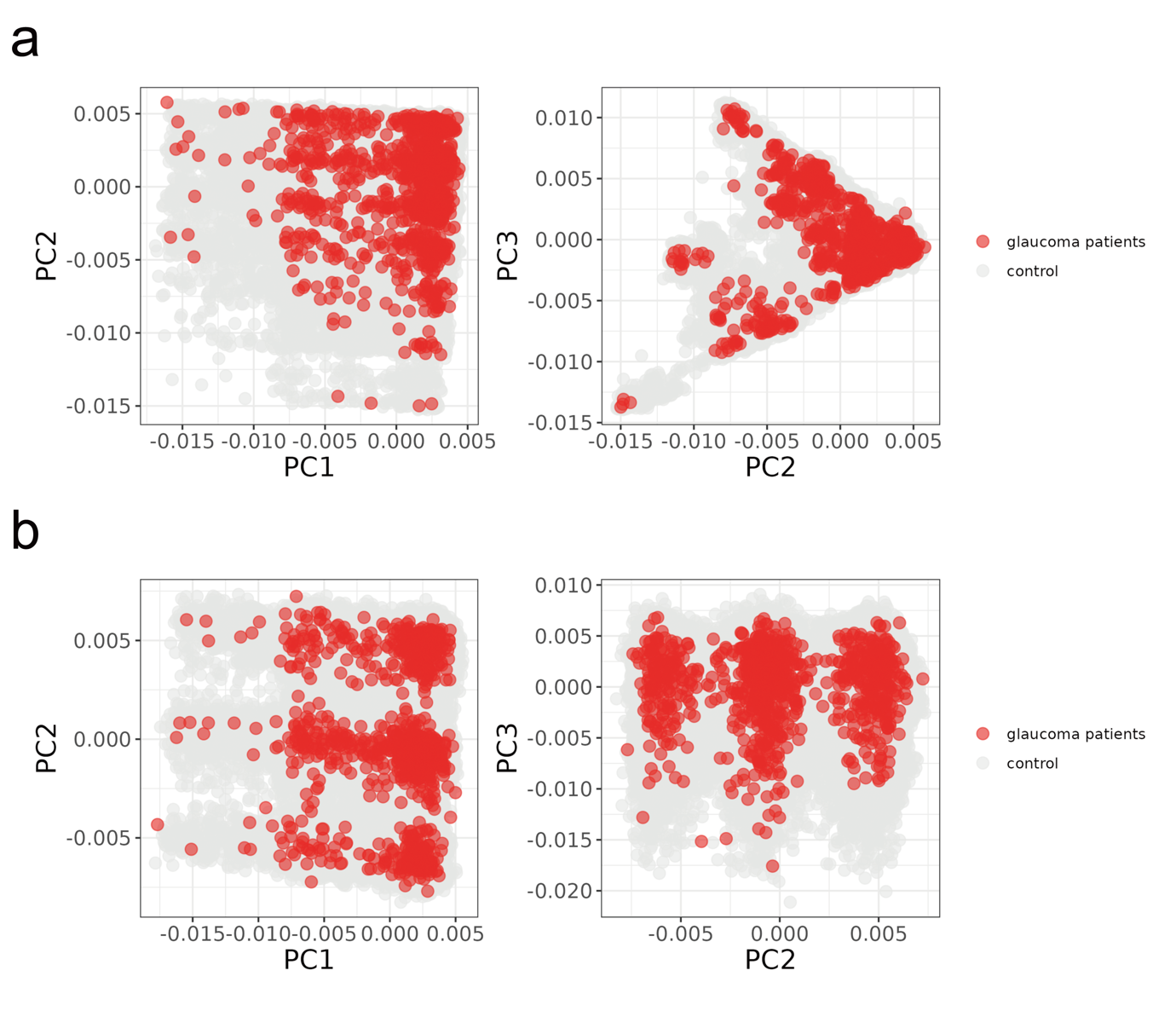


**Supplementary Fig. S6.** Principal component analysis (PCA) of genetic variants in 82,147 TLCPD-estimated UKBB participants of European ancestry. The first three principal components of genetic ancestry are presented. Each dot represents a TLCPD-estimated UKBB participant of European ancestry. **a**, PCA using variants with MAF ≥ 0·05 and LD *r*^2^ < 0·5 between each pair of variants. **b** PCA using variants further restricted to HapMap3 variants. PC, principal component; PCA, principal component analysis; TLCPD, trans-laminar cribrosa pressure difference; UKBB, UK Biobank; MAF, minor allele frequency; LD, linkage disequilibrium.

**
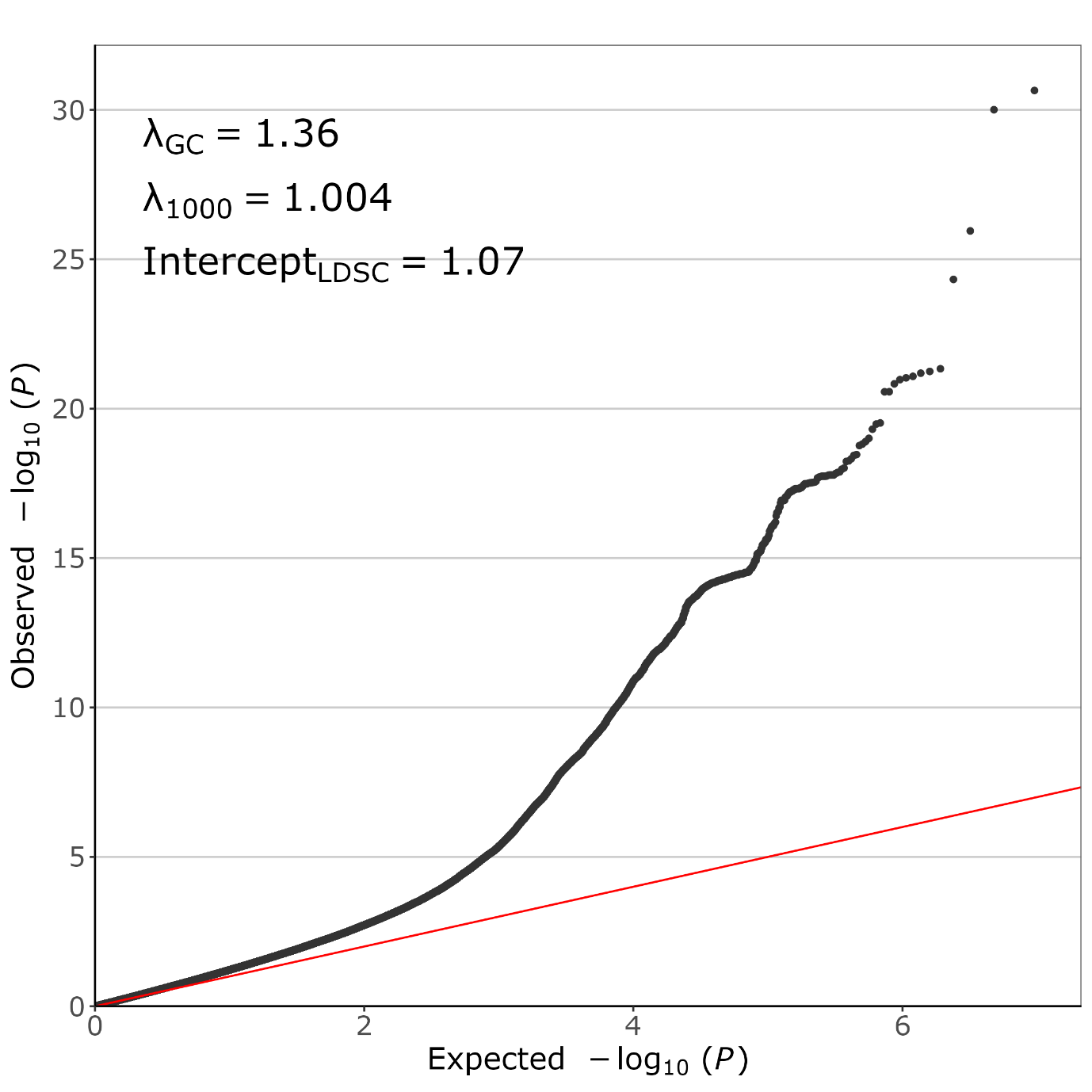
**

**Supplementary Fig. S7.** Quantile-quantile plot for GWAS of TLCPD (n = 82,147). The quantile-quantile plot displays the relationship between the observed *P*-values (*y*-axis) and the expected *P-*values of a null distribution (*x*-axis) on a −log_10_ scale. The genomic inflation factor (λ_GC_), the scaled genomic inflation factor for a sample size of 1,000 (λ_1000_), and the LDSC intercept are presented in the upper left corner to demonstrate the absence of significant population stratification. GWAS, genome-wide association study; TLCPD, trans-laminar cribrosa pressure difference; GC, genomic control; LDSC, linkage disequilibrium score regression; *P*, *P*-value.

**
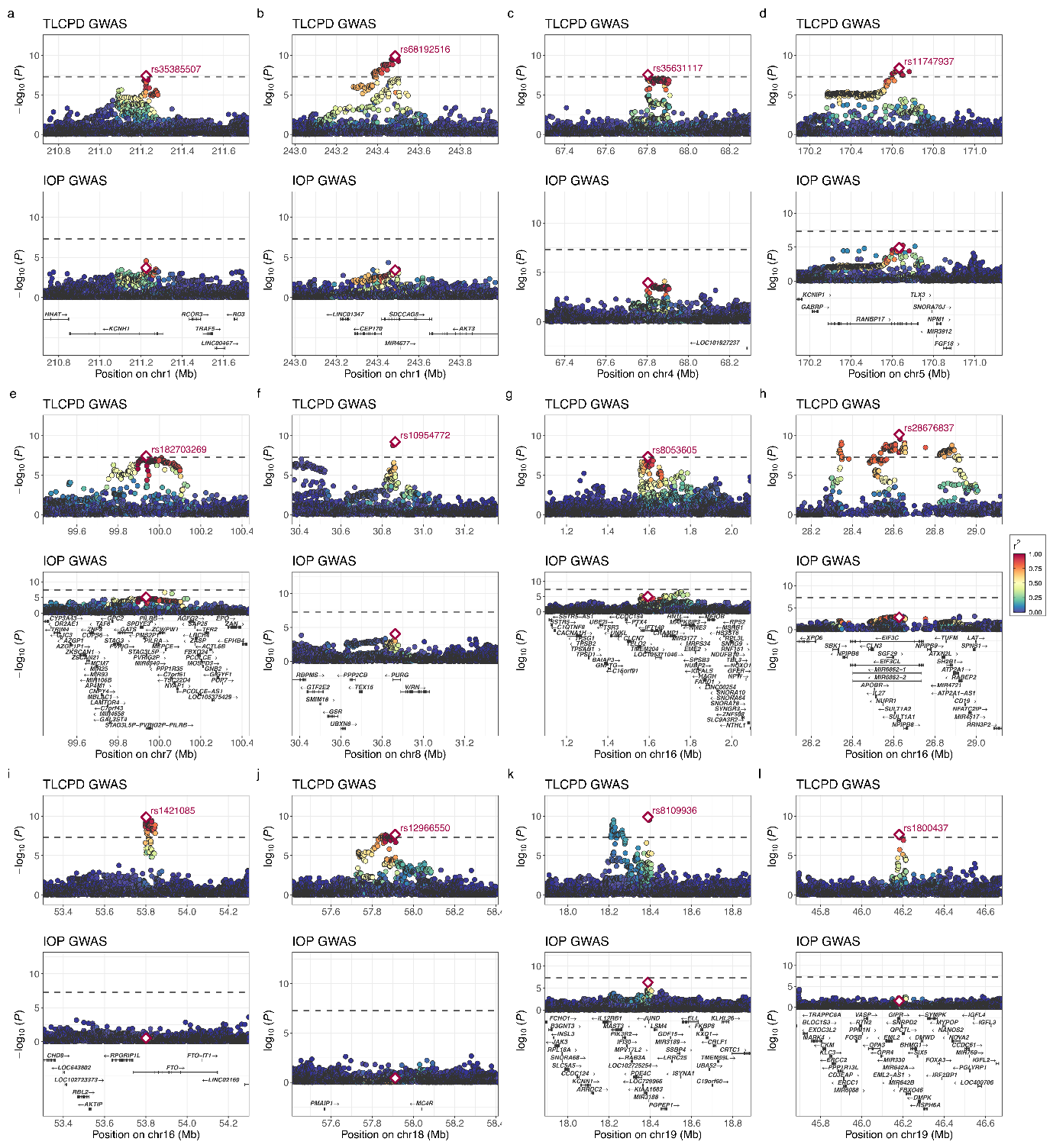
**

**Supplementary Fig. S8.** Regional plots of previously unreported loci in the TLCPD GWAS (top) and IOP GWAS (bottom). **a-l**, Regional plots of previously unreported loci in the TLCPD GWAS (top) and IOP GWAS (bottom). The plots are colored based on different lead variants, displaying ±500 kb regions from the lead variant in each locus. Each dot represents a variant plotted as –log_10_ (*P*) on the *y*-axis against the corresponding variant position (Mb) on the *x*-axis, and the variants are colored according to linkage disequilibrium with the lead variant (rhombus). TLCPD, trans-laminar cribrosa pressure difference; GWAS, genome-wide association study; IOP, intraocular pressure; *P*, *P*-value.

**
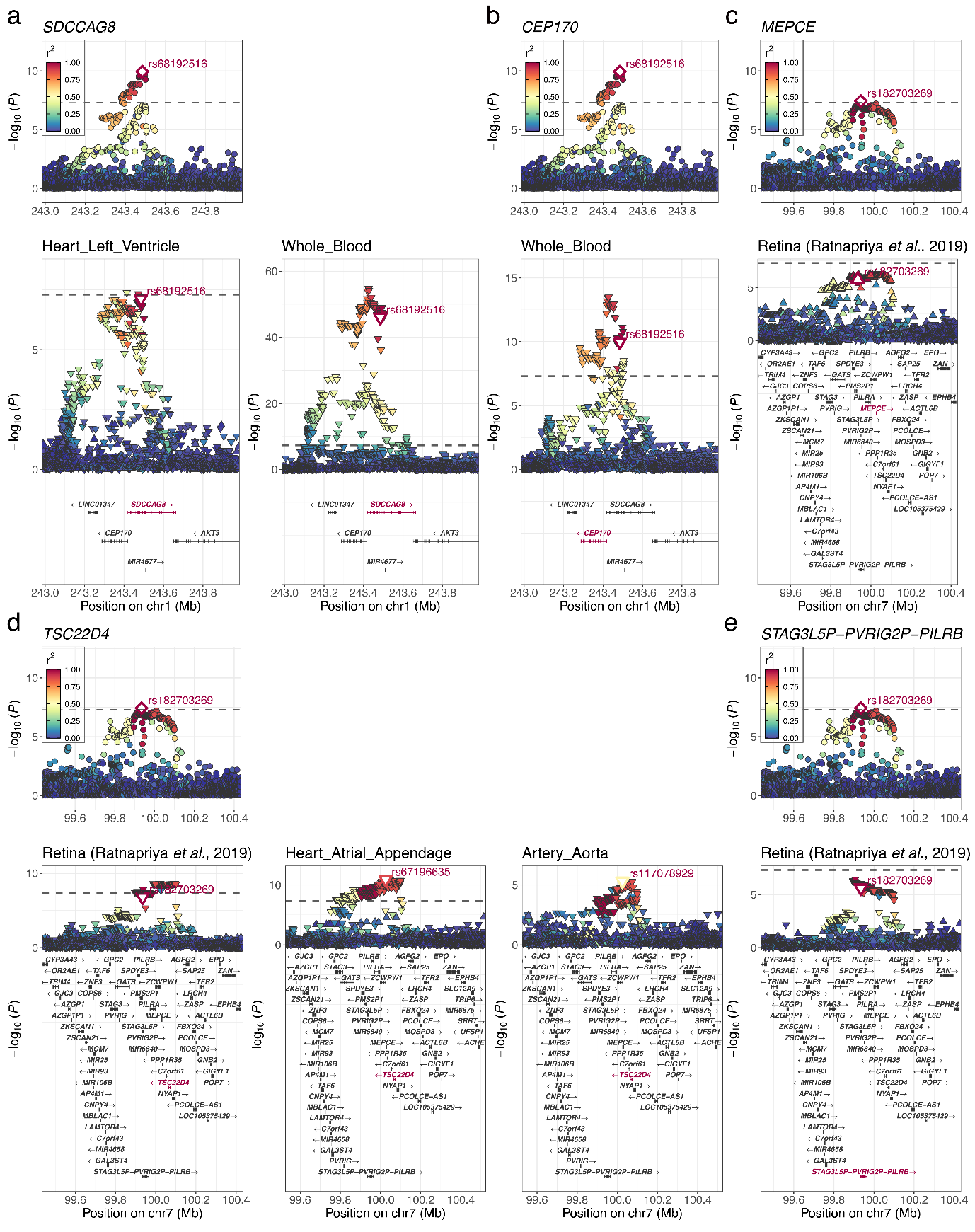

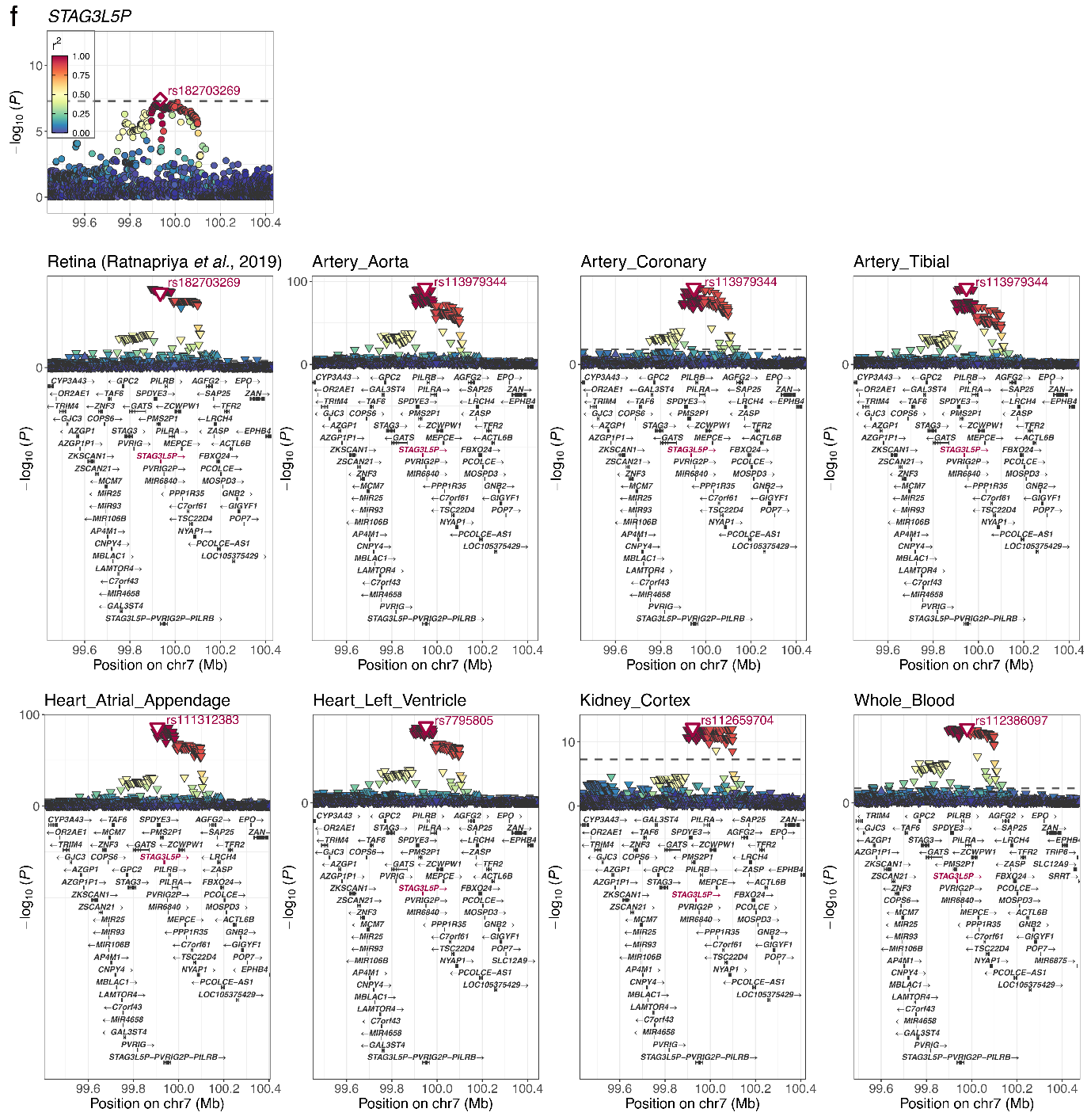

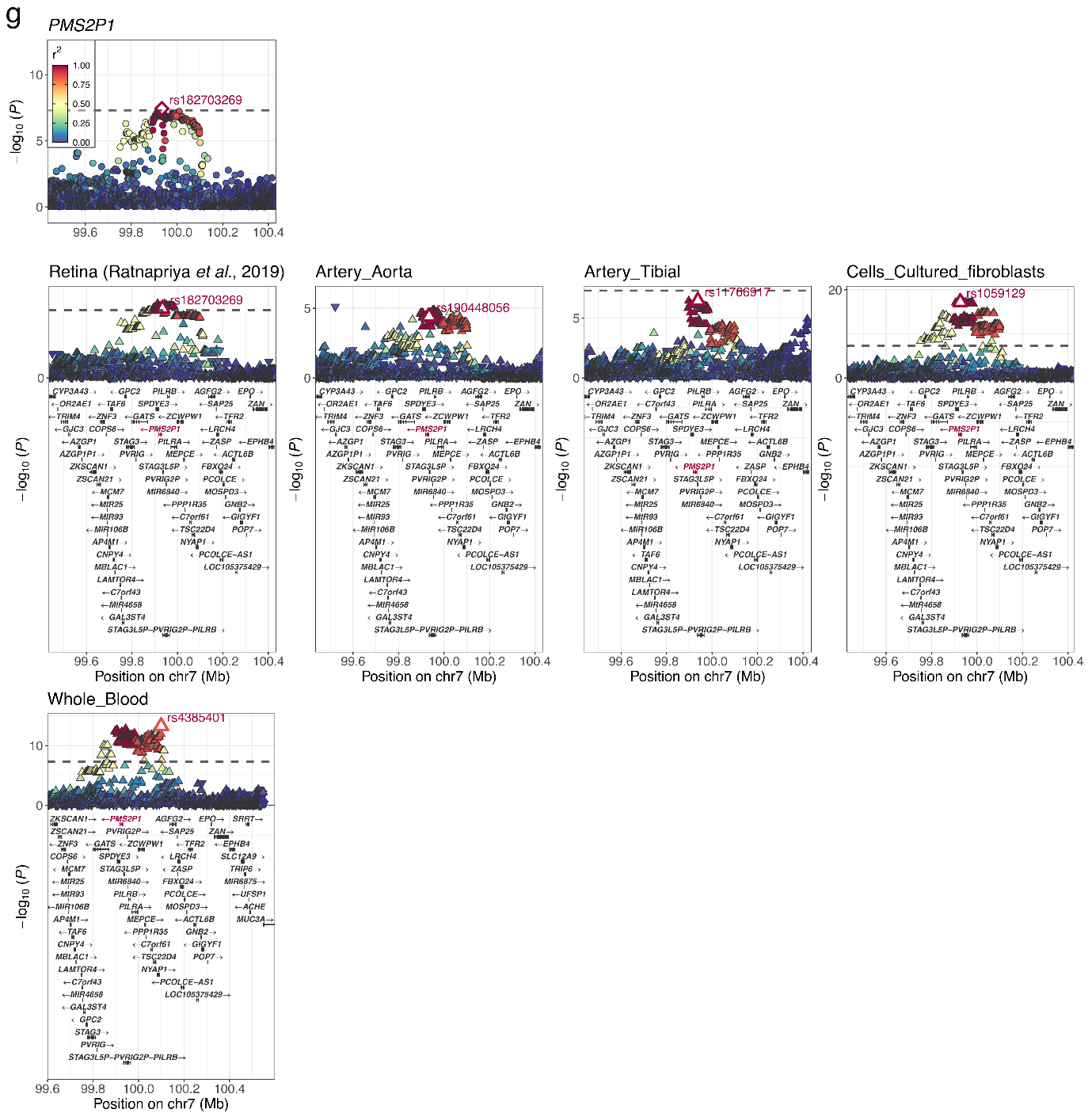

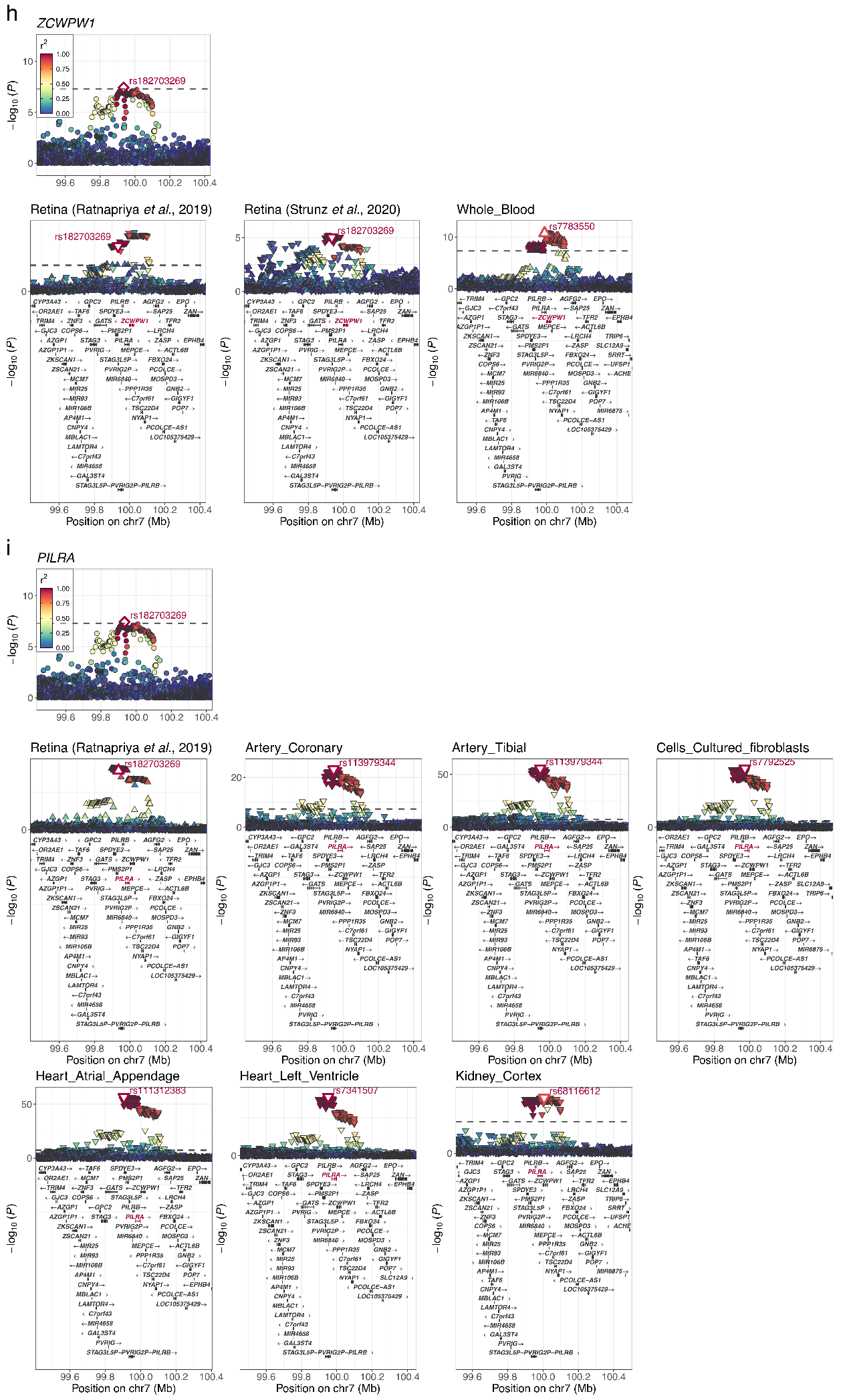

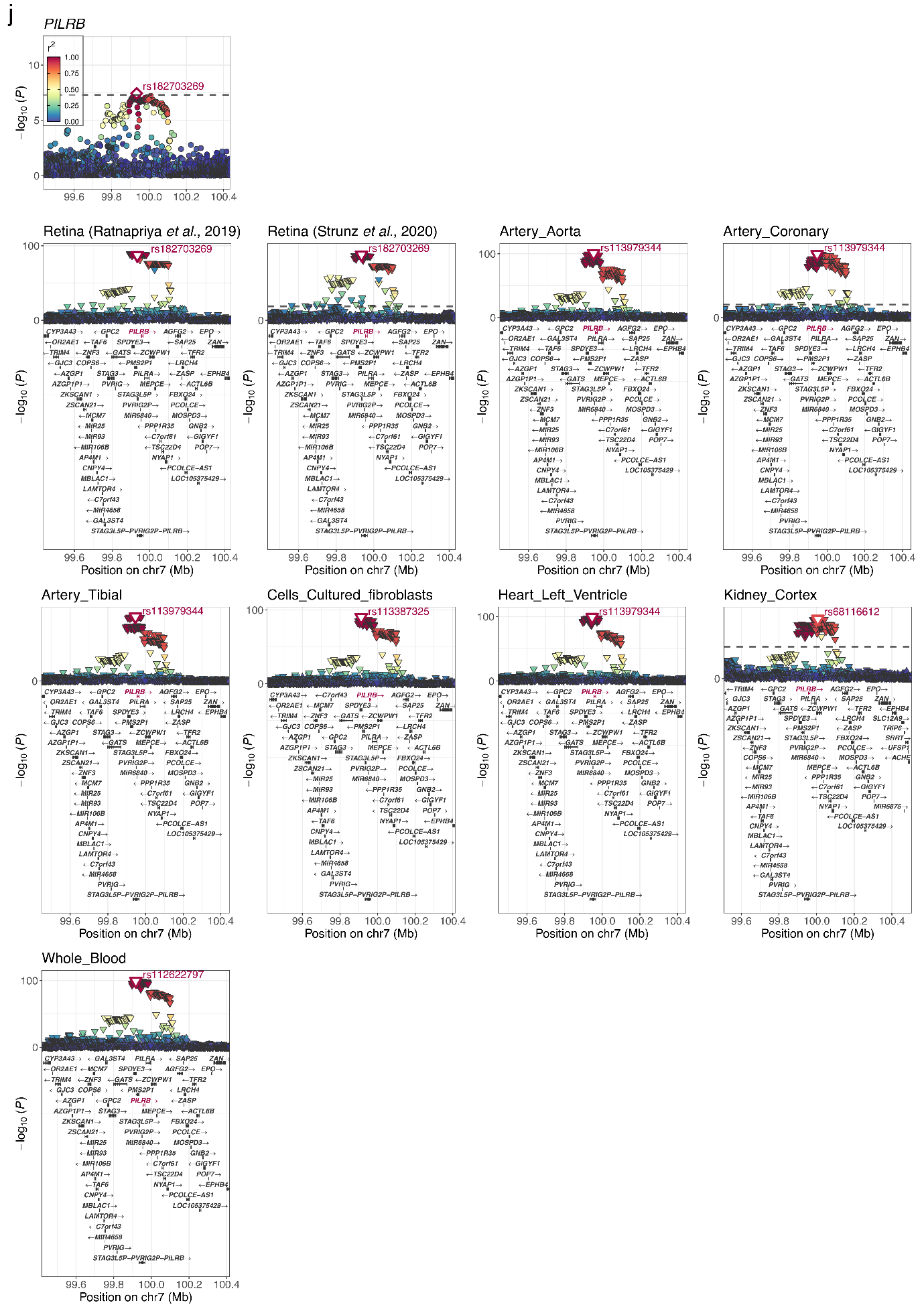

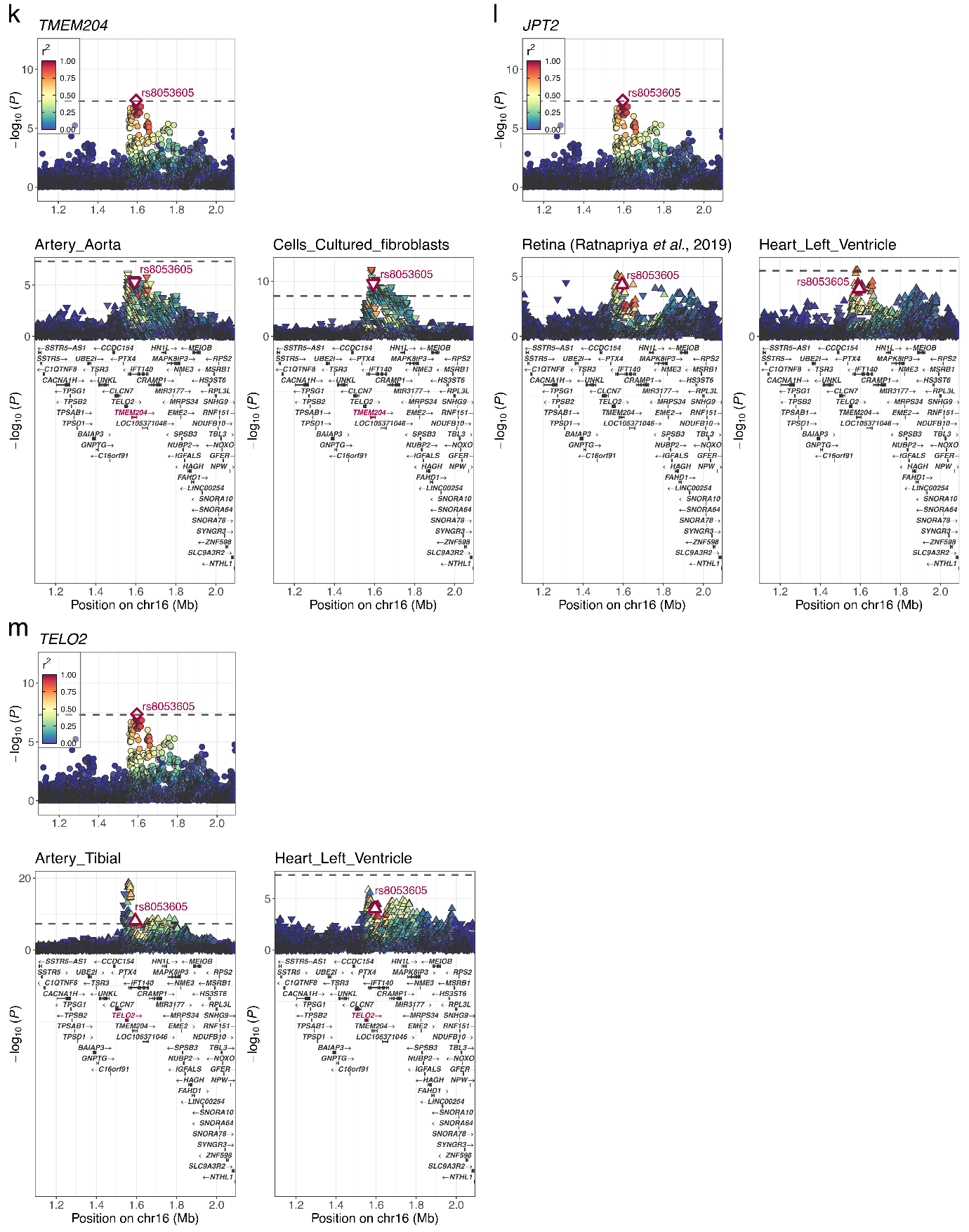

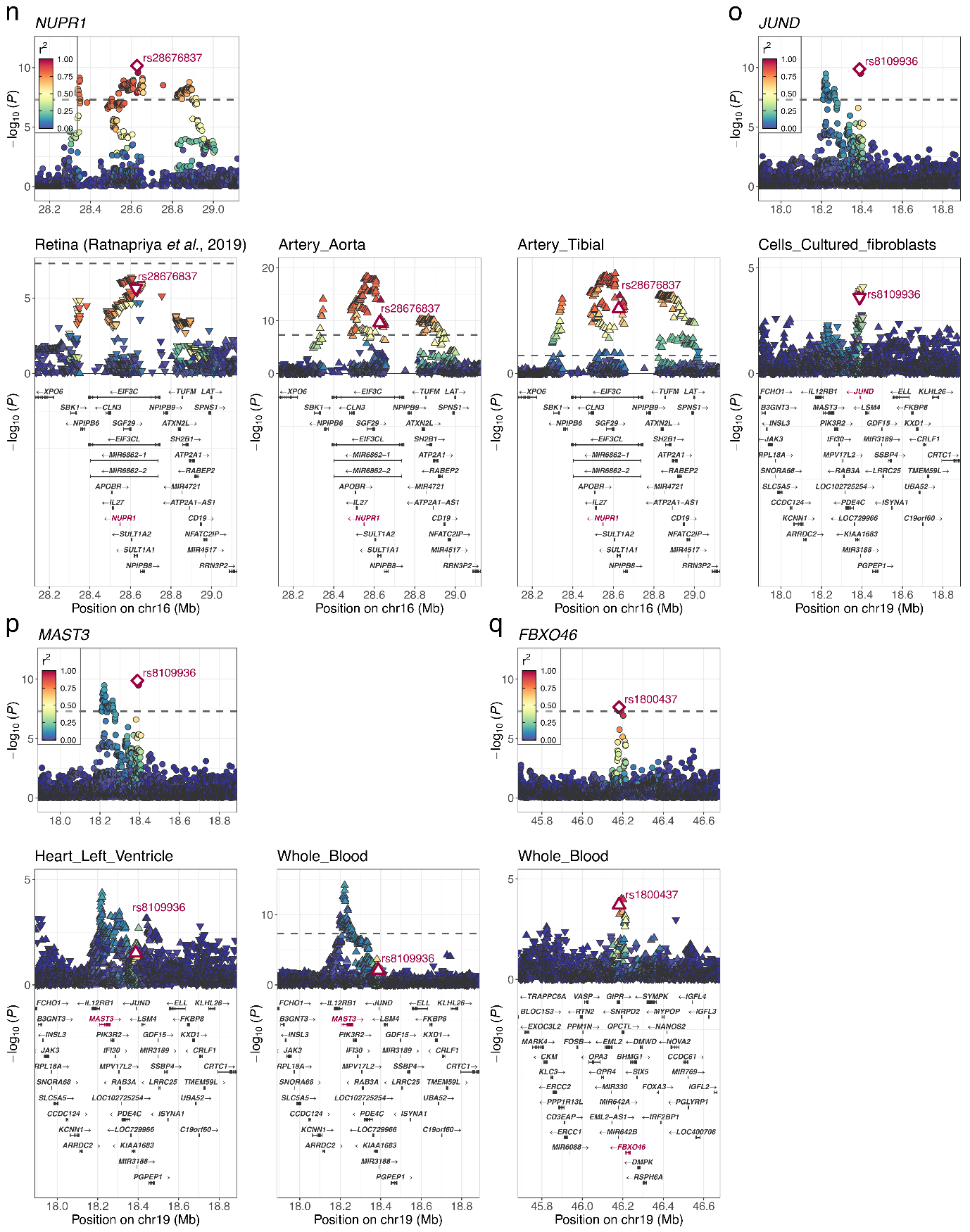
**

**Supplementary Fig. S9.** Regional plots of previously unreported and colocalized loci from TLCPD GWAS (top) and eQTLs of the colocalized gene (bottom). **a-q,** regional plots of previously unreported and colocalized loci from TLCPD GWAS (top) and eQTLs of the colocalized gene (bottom). The plots are displaying ±500kb regions from the colocalized (PP.H4 ≥ 0·8) variant in each locus. Each dot represents a variant plotted as –log_10_ (*P*) on *y*-axis against the corresponding variant position (Mb) on the *x*-axis. Variants are colored according to linkage disequilibrium with the lead variant (rhombus) or the variant nearest to the lead variant if it does not exist in eQTL (triangle). PP.H4, posterior probability for colocalization; TLCPD, trans-laminar cribrosa pressure difference; GWAS, genome-wide association study; eQTL, expression quantitative trait loci; *P*, *P*-value.


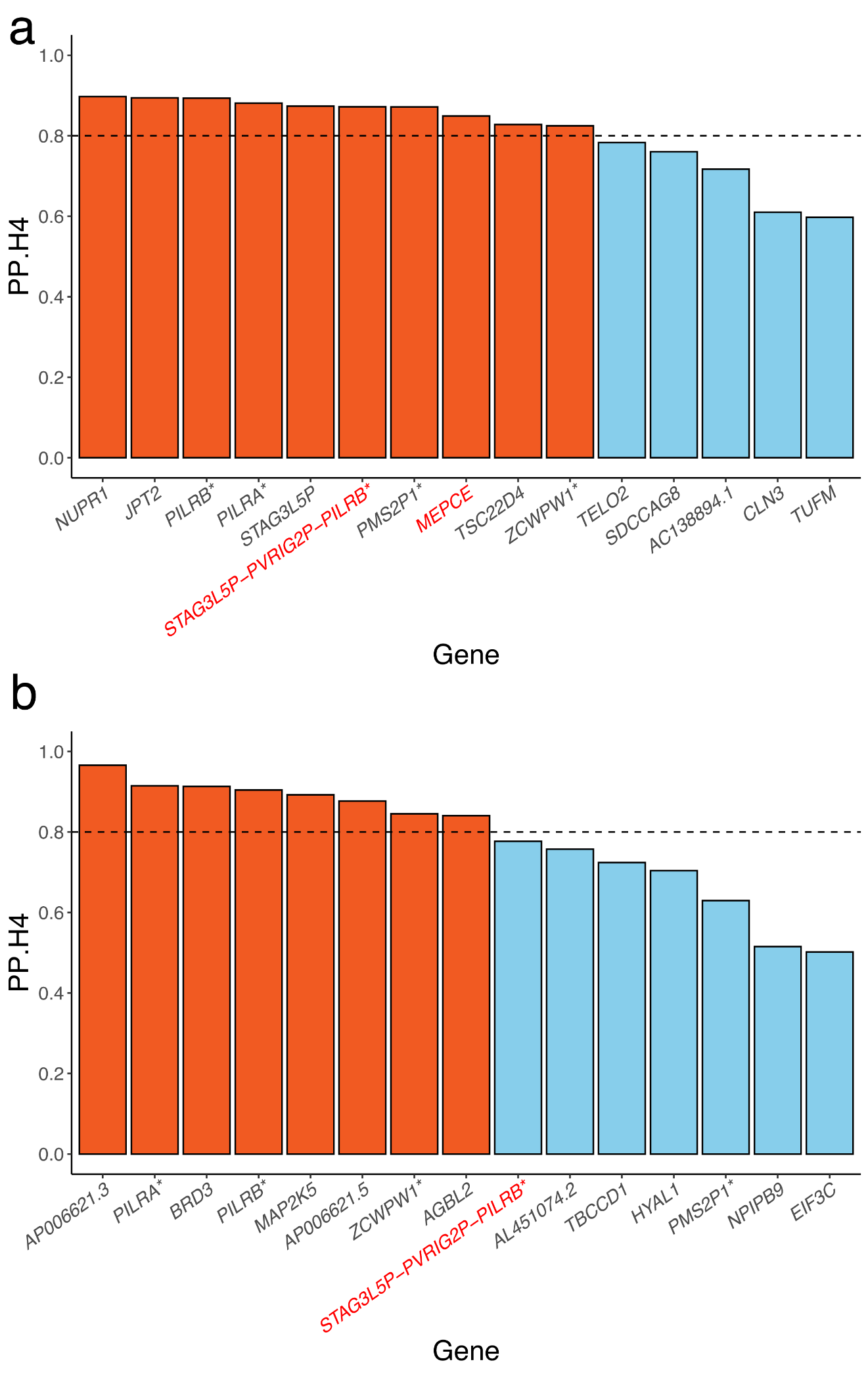


**Supplementary Fig. S10.** Bar plot of PP.H4 values in retinal tissues for genes colocalized with unreported loci. **a,** bars represent the posterior probability of each gene for H4 (PP.H4) in retinal tissue of EyeGEx (Ratnapriya et al., 2019), indicating that both traits were associated with shared causal variants. Common genes in both retinal colocalization results were highlighted with asterisk. Red genes represent colocalized genes in previously unreported loci, exclusively in retinal tissue. Gray dashed line represents the colocalization threshold (PP.H4 = 0·8). Orange bars indicate significant colocalization that passed the colocalization threshold. **b,** bars represent the posterior probability of each gene for H4 (PP.H4) in retinal tissue from Strunz et al., 2020, indicating that both traits were associated with shared causal variants. Common genes in both retinal colocalization results were highlighted with asterisk. Red genes represent colocalized genes in previously unreported loci, exclusively in retinal tissue. Gray dashed line represents the colocalization threshold (PP.H4 = 0.8). Orange bars indicate significant colocalization that passed the colocalization threshold. PP.H4, posterior probability for colocalization.

**References**

1. Xie X, Zhang X, Fu J, et al. Noninvasive intracranial pressure estimation by orbital subarachnoid space measurement: the Beijing Intracranial and Intraocular Pressure (iCOP) study. *Crit Care*. Jul 24 2013;17(4):R162. doi:10.1186/cc12841

2. Fleischman D, Bicket AK, Stinnett SS, et al. Analysis of Cerebrospinal Fluid Pressure Estimation Using Formulae Derived From Clinical Data. *Invest Ophthalmol Vis Sci*. Oct 1 2016;57(13):5625-5630. doi:10.1167/iovs.16-20119

3. O'Connell J, Sharp K, Shrine N, et al. Haplotype estimation for biobank-scale data sets. *Nat Genet*. Jul 2016;48(7):817-820. doi:10.1038/ng.3583

4. Howie BN, Donnelly P, Marchini J. A flexible and accurate genotype imputation method for the next generation of genome-wide association studies. *PLoS Genet*. Jun 2009;5(6):e1000529. doi:10.1371/journal.pgen.1000529

5. Yang J, Lee SH, Goddard ME, Visscher PM. GCTA: a tool for genome-wide complex trait analysis. *Am J Hum Genet*. Jan 7 2011;88(1):76-82. doi:10.1016/j.ajhg.2010.11.011

6. Freedman ML, Reich D, Penney KL, et al. Assessing the impact of population stratification on genetic association studies. *Nat Genet*. Apr 2004;36(4):388-393. doi:10.1038/ng1333

7. Bulik-Sullivan BK, Loh PR, Finucane HK, et al. LD Score regression distinguishes confounding from polygenicity in genome-wide association studies. *Nat Genet*. Mar 2015;47(3):291-295. doi:10.1038/ng.3211

8. Bulik-Sullivan B, Finucane HK, Anttila V, et al. An atlas of genetic correlations across human diseases and traits. *Nat Genet*. Nov 2015;47(11):1236-1241. doi:10.1038/ng.3406

9. Genomes Project C, Auton A, Brooks LD, et al. A global reference for human genetic variation. *Nature*. Oct 1 2015;526(7571):68-74. doi:10.1038/nature15393

10. Gharahkhani P, Jorgenson E, Hysi P, et al. Genome-wide meta-analysis identifies 127 open-angle glaucoma loci with consistent effect across ancestries. *Nat Commun*. Feb 24 2021;12(1):1258. doi:10.1038/s41467-020-20851-4

11. Zeng J, Xue A, Jiang L, et al. Widespread signatures of natural selection across human complex traits and functional genomic categories. *Nat Commun*. Feb 19 2021;12(1):1164. doi:10.1038/s41467-021-21446-3

12. Prive F, Arbel J, Vilhjalmsson BJ. LDpred2: better, faster, stronger. *Bioinformatics*. Apr 1 2021;36(22-23):5424-5431. doi:10.1093/bioinformatics/btaa1029

13. de Leeuw CA, Mooij JM, Heskes T, Posthuma D. MAGMA: generalized gene-set analysis of GWAS data. *PLoS Comput Biol*. Apr 2015;11(4):e1004219. doi:10.1371/journal.pcbi.1004219
